# Artificial intelligence in financial market prediction: advancements in machine learning for stock price forecasting

**DOI:** 10.3389/frai.2025.1696423

**Published:** 2026-01-13

**Authors:** Arafat Rohan, Md. Deluar Hossen, Md. Nuruzzaman Pranto, Balayet Hossain, Areyfin Mohammed Yoshi, Rakibul Islam

**Affiliations:** International American University, Los Angeles, CA, United States

**Keywords:** artificial intelligence, financial market, machine learning, market prediction, risk management, stock market

## Abstract

This study reviews the advancements in AI-driven methods for predicting stock prices, tracing their evolution from traditional approaches to modern finance. The role of AI in the market extends beyond predictive systems to encompass the intersection of financial markets with emerging technologies, such as blockchain, and the potential influence of quantum computing on economic modeling. A decentralized finance system examines the application of Reinforcement Learning in financial market prediction, highlighting its potential for continuous learning from dynamic market conditions. The study discusses the development of hybrid prediction models, stock market machine learning systems, and AI-driven investment portfolio management. The potential of quantum computing enhances portfolio analysis, fraud detection, optimization, and asset valuation for complex market predictions, as well as the impact of blockchain technologies on transparency, security, and efficiency. Machine learning techniques can significantly automate data collection and purification. Financial decision-making and the application of time-series analysis techniques can be readily learned through deep reinforcement learning for stock price prediction. Deep Neural Networks and Strategic Asset Allocation can be managed by evaluating performance and portfolio using real-time market insights from AI models. Although there are numerous ethical, sentimental, regulatory, and data quality issues in market prediction, the future job market is heavily dependent on these criteria, particularly through effective risk management and fraud detection.

## 1 Introduction

Over the past several decades, the field of finance has undergone a significant evolution, driven mainly by the introduction of Machine Learning (ML) and Artificial Intelligence (AI). Artificial intelligence has not only altered trading and investing strategies but also impacted financial market forecasts. Financial firms are actively developing analytical models to provide more accurate estimates of stock market values, leveraging vast volumes of data and intricate computations (Xiao and Ke, 2021; Rouf et al., 2021). Economic indicators and outdated mathematical models were previously used to make predictions in the financial markets sector. To make accurate forecasts of stock prices, significant effort would be invested in analyzing financial data and macroeconomic indicators using mathematical models and formulas. Despite providing some insight into the issue, these methods were insensitive to changing market conditions and were unable to handle semi-structured and unstructured variables. With the use of AI and machine learning, practice will go beyond these approaches in the future, providing designers with more options for analyzing market conditions (Kumar et al., 2020; Ahmed et al., 2022; Ndikum, 2020). Machine learning (ML) is particularly well-suited for analyzing stock market data due to its adaptability and learning capabilities. Artificial intelligence is a broader concept, and machine learning is one of its types that enables predicting the state of financial markets by using computers to train them. Standard techniques, such as supervision, unsupervision, and reinforcement, are used to increase the predictability of stock price values. For example, supervised learning algorithms utilize data to train models that can then forecast future stock prices when new inputs are introduced (Kurani et al., 2021; Nabipour et al., 2020).

Data is at the core of machine learning, particularly in the financial markets. Data sources include unformatted information from newspapers and social media, as well as comparable economic statistics and formatted data based on past pricing (Vijh et al., 2020). More insight into market trends is possible thanks to NLP technology, which also helps the machine learning algorithm derive context and sentiment from text blocks. Since it provides a more comprehensive perspective on potential pricing, combining multiple data sources enhances the accuracy of the prediction models' entries (Kamalov, 2020). The machine learning approaches used to forecast stock prices rely on a variety of algorithms. Among the most often used methods are random forests, gradient boosting, decision trees, and neural networks (Alshater et al., 2022). Deep learning models are neural networks that are widely used and have gained popularity for their ability to learn hierarchical representations from data. These methods make it feasible to use non-linear stock price relationships, which leads to the assumption of linear models and, therefore, to the formulation of accurate forecasts (Fathali et al., 2022; Aldhyani and Alzahrani, 2022). The use of large datasets and advances in computing power significantly enhance the effectiveness of machine learning in the financial industry. There is considerable room for real-time processing due to the massive data volume processed per second in financial markets. Cloud computing and graphics processing units (GPUs) are two powerful computing resources that can be used to handle large datasets, also known as “big data,” thereby improving the accuracy of algorithms (Biju and Thomas, 2023; Shah et al., 2022).

AI can be used to anticipate market trends in various financial domains, including intraday computations and securities investments. To capitalize on micro price fluctuations, they also facilitate transactions at frequencies and speeds that exceed human capabilities to replicate (Kumar et al., 2021). Additionally, many investment companies use machine learning to enhance decision-making for investment portfolios and better manage the associated risk factors. Based on past performance and market conditions, these models may provide guidance on where to buy or sell, thereby enhancing the decision-making process (Rane N. L. et al., 2024). Nevertheless, several challenges exist in utilizing AI and machine learning for financial market forecasting. Overfitting, a situation where the introduced model becomes overly complex and begins selecting arbitrary patterns in the data rather than the actual signals, is another challenge.

Furthermore, specific machine learning algorithms are referred to as “black boxes.” For practitioners, this makes it challenging to interpret model results and identify the elements that influenced the ultimate choice. Other regulatory issues arise because financial organizations must comply with laws regarding the use of data and algorithms for trading (Bahoo et al., 2024; Nti et al., 2019; Hamayel and Owda, 2021). Ethical questions about market fairness arise from the use of AI in finance. Because they can execute deals at such high speeds, high-frequency trading algorithms have the potential to exacerbate volatility or even cause flash crashes. To prevent prejudice, data management must also be conducted with consideration for potential biases. To ensure that frameworks for the various systems that use AI are established, it is essential to understand their ethical implications (Bhandari et al., 2022; Rezaei et al., 2020; Bustos and Pomares-Quimbaya, 2020). The extent to which AI is used in financial market prediction may improve further in the future. Better forecasting and the release of models combining AI and economic theories are predicted to result from the continued development of machine learning techniques (Muhammad et al., 2023). Moreover, the growing volume of external data, which is not necessarily generated within the automobile, such as satellite photos and consumers' transaction history, will provide additional inputs to AI models, enhancing their insights and, thus, the quality of the judgments made (Pattnaik et al., 2023; Ayitey Junior et al., 2023). To improve stock price forecasting, recent advances in artificial intelligence for financial market prediction have focused on combining deep learning systems, such as transformer-based systems and graph neural networks, with other data sources, including sentiment analysis from social media, satellite imagery, and real-time news analytics (Sonkavde et al., 2023). To maximize strategy adaptation and be honest about choices, the models investigate the benefits of hybrid approaches that combine explainable AI techniques with reinforcement learning and conventional technical and fundamental indicators. Stronger, more reliable, and more adaptable (adaptive quantity) market prediction systems are enabled by federated learning and quantum machine learning, which also enable faster computations and privacy-preserving model training on decentralized financial data (Chang et al., 2024; Najem et al., 2024).

## 2 Overview of financial market prediction

This review employed a systematic, structured literature search strategy to ensure comprehensive, unbiased coverage of studies on AI-driven financial market forecasting. Four major academic databases were used: Scopus, Web of Science, IEEE Xplore, and Google Scholar, as these platforms index high-impact journals and conferences in computer science, finance, and data science. The search covered the period 2018–2024, capturing the most recent developments in machine learning, deep learning, reinforcement learning, blockchain analytics, and quantum computing applications in financial prediction.

The following keywords and Boolean combinations were used during the search: “AI in finance,” “stock market prediction,” “machine learning forecasting,” “deep learning stock price prediction,” “reinforcement learning trading,” “hybrid prediction models,” “sentiment analysis stock forecasting,” “blockchain financial analytics,” and “quantum computing finance.” These keyword sets were refined iteratively to capture both domain-specific and cross-disciplinary research.

Financial market prediction is the process of forecasting future market movements, asset prices, and economic outlooks using statistical models and analytical procedures, often in conjunction with machine learning algorithms. For equities, commodities, currency, and cryptocurrencies, it combines sentiment research, technical analysis, fundamental analysis, and historical integration (Thakkar and Chaudhari, 2020). Traditional approaches include econometric, regression, and time-series analysis. To improve accuracy, modern techniques leverage artificial intelligence, deep learning, and big data analytics (Cavalcante et al., 2016). These impacts (influences) are forecast in several ways, including market mood, investor behavior, geopolitical developments, and macroeconomic statistics. Despite these developments, market forecasting remains problematic due to human unpredictability, exogenous shocks, and volatility (Kumar et al., 2021).

One of the most extensively researched and challenging problems is stock price prediction, which attracts scholars from diverse disciplines, including business, mathematics, computational science, and economics. Since stock price prediction can yield substantial gains, it has been a focus of attention for years (Shah et al., 2019). Due to the near-random nature of stock time series, stock market prediction is a challenging endeavor. Due to its unpredictability, stock market forecasting is one of the most challenging undertakings. Even a slight improvement in the new algorithm's predictions can yield significant earnings, as the stock market prediction challenge is highly lucrative. A crucial component of the forecast is the price of stocks (Nti et al., 2019). The rapidly expanding financial markets of recent years have provided investors with new options while also presenting new challenges for financial analysts seeking to mitigate investment risks and make informed decisions (Chakraborti et al., 2007). Because numerous interrelated factors influence future pricing, the stock market is a highly complex and dynamic system. The idea that financial markets are predictable has been the subject of intense research (Nabipour et al., 2020). Investors may now access stock markets more efficiently, thanks to technological advancements. A variety of techniques, including machine learning, data mining, and statistical models, have been proposed for stock market prediction in both industry and academia (Kumbure et al., 2022). Over time, specific long-term hypotheses about market exchanges have been developed. They either attempt to explain what market exchanges are or discuss whether company sectors can be outperformed (Nassirtoussi et al., 2014). Although traders and financial institutions have developed a variety of models to outperform the market for their clients or themselves, only a few have consistently achieved higher-than-average profitability. Machine learning can forecast the stock market by utilizing historical datasets, social media data, and financial news or trends to train and test models (Raj et al., 2022).

### 2.1 The historical context of financial market analysis

A fascinating path characterized by the growing integration of technology can be traced back to the history of financial market analysis, which has culminated in the contemporary period dominated by artificial intelligence (AI). Traditionally, fundamental analysis, which focuses on industry circumstances, business financials, and macroeconomic indicators, has been the primary basis for investment decisions (Tulsyan et al., 2024). For many years, this method, based on human knowledge and intuition, was the cornerstone of investment strategies. However, the introduction of computers and quantitative finance in the late twentieth century brought about a paradigm change. As a supplementary technique, technical analysis employs statistical models to analyze historical market data and forecast future price movements (Tay and Shen, 2002). The foundation for the digital revolution in financial markets was laid during this time by the creation of complex algorithms capable of handling enormous volumes of data at previously unheard-of speeds. High-frequency trading, or HFT, became popular around the turn of the century. In HFT, algorithms execute deals at the best times in milliseconds. Thanks to developments in network infrastructure and processing capacity, this was a significant step forward in the automation of trading (Vázsonyi, 2009). Because they can operate at scales and speeds that humans cannot, HFT algorithms have played a crucial role in shaping market characteristics, including volatility and liquidity (Weng et al., 2017). A new era of data-driven decision-making in finance began as we entered the second decade of the twenty-first century, marked by the emergence of big data and machine learning, which sparked yet another wave of innovation in financial market analysis (Jena et al., 2023). Artificial intelligence (AI) and intense learning have begun to play a crucial role in identifying intricate patterns within large datasets, such as news articles, economic indicators, and social media sentiment. These models, trained on historical data, were able to forecast stock prices more accurately than traditional methods (Fang et al., 2014). The continuous search for more precise and effective forecasting models is evident in the historical shift from fundamental and technical analysis to the current AI-driven era. Every stage has improved on the one before it, using new technology while maintaining the fundamentals of market analysis. AI is now leading the way, providing unmatched insights into investor behavior and market patterns and laying the groundwork for future financial market forecasting. AI has the potential to significantly transform risk management, portfolio optimization, and investment methods as it continues to develop (Thakor, 1996). Understanding the historical background of financial market research is crucial for comprehending the relevance of AI in stock market prediction today and the potential for further advancements in this rapidly evolving sector (Sahu et al., 2023). One of the more inventive features is the reanalysis of historical market data using behavioral finance principles to show how previously ignored early market behavior patterns can be quantitatively compared with available data to identify investor cyclical behavior. Despite technological advances, certain inefficiencies persist, and the defined technique highlights the underappreciated parallels between early decision-making and the current algorithmic trading process (Rouf et al., 2021; Kurani et al., 2021).

**Figure 1:**
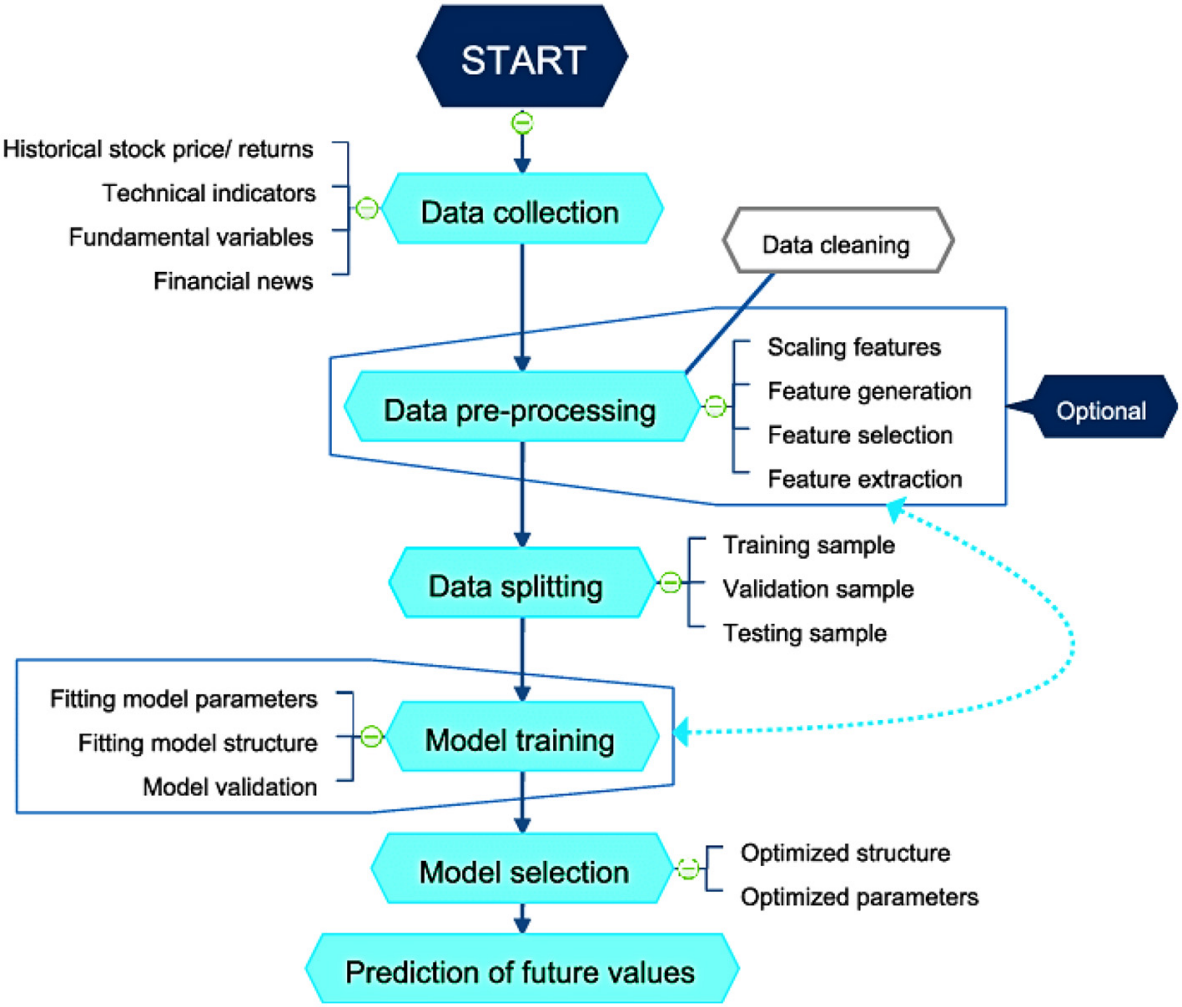
Process of a supervised learning stock market prediction model (Kumbure et al., 2022).

Figure 1 illustrates the process for a supervised learning-based stock market prediction model. It begins with data collection and proceeds to technical indicators, fundamental factors, historical stock prices, and financial news. The next step in the data preparation process is optional feature extraction, followed by scaling, cleaning, and feature generation. After that, all of this is divided into test, validation, and training samples. The model training step involves fitting the model parameters, organizing the model, and then evaluating its performance. The model selection process will train the structure and parameters to maximize accuracy. Next, the trained model anticipates future values, which also forecasts the stock market's movements. An iterative procedure for enhancing model performance and generating reliable market predictions is illustrated in this figure.

### 2.2 Modern finance and the role of technology

Technology plays a complex and revolutionary role in contemporary finance, profoundly changing the structure of financial markets and investment strategies. Technology is a driving force behind innovation, efficiency, and accessibility in the modern world, changing the way financial services are provided and used (Hsu et al., 2016). The digitalization of financial services is a central component of this change. The way investors obtain information, make trades, and oversee their portfolios has been completely transformed by digital platforms. Robotic advisers, smartphone banking applications, and online trading platforms have democratized financial services by giving regular people access to resources previously available only to large investors (Kirtac and Germano, 2025). These platforms utilize advanced algorithms to automate trading, optimize portfolio management, and provide personalized financial advice, all while reducing transaction costs and enhancing market participation.

Furthermore, traditional banking and financial services have been impacted by the emergence of fintech (financial technology) enterprises (Trinh, 2025). Fin-tech companies have launched new goods and services that enhance the consumer experience, optimize processes, and mitigate risks by leveraging cutting-edge technologies such as blockchain, cloud computing, and artificial intelligence (Qi et al., 2024). Blockchain technology, for example, can completely transform how financial transactions are handled by enabling faster settlement times and lower fraud risk. In a similar vein, cloud computing enables scalable, cost-effective storage and analysis of data, thereby improving operational efficiency and decision-making (Rahmouni, 2025). Mainly, AI has been a game-changer in contemporary finance. Real-time processing of enormous volumes of data, pattern recognition, and remarkably accurate prediction are all possible with AI-powered systems (Dakalbab et al., 2024). This capacity is utilized in various financial sectors, including credit scoring, fraud detection, portfolio management, and algorithmic trading. To anticipate stock prices and assess market sentiment, artificial intelligence (AI) models, particularly those based on deep learning, can analyze large, complex datasets, such as text from news articles and social media (Che C. et al., 2024).

Additionally, artificial intelligence (AI) is driving advancements in Natural Language Processing (NLP), enabling robots to comprehend and interpret human language (Najem et al., 2024). The integration of AI with traditional financial models through hybrid approaches has proven particularly effective. These models combine the strengths of AI with established financial theories, such as the Capital Asset Pricing Model (CAPM) and the Efficient Market Hypothesis (EMH), to create more robust predictive frameworks that better account for market anomalies and irrational investor behavior, leading to improved investment outcomes (Kumar et al., 2024). Natural language processing (NLP) is crucial for sentiment analysis, where algorithms analyze social media and news feeds to gauge public opinion on specific stocks or economic events, providing valuable insights for informed trading strategies (Tashakkori et al., 2024). The novelty lies in the synchronization of powerful machine learning models with advanced high-frequency trading platforms. This enables new decision-making to adapt to sudden changes in the market regime, while also increasing speed. Unlike earlier automation methods that relied on a fixed, rule-based platform, these models can retrain at a streaming level in near real-time and combine self-maintaining algorithms with financial theory to increase their resilience in highly volatile markets (Zhang et al., 2023; Chhajer et al., 2021).

### 2.3 A system for predicting the stock market has been proposed

Combining machine learning techniques with financial data processing and computational models enables a stock market prediction system to function effectively. Information gathering from historical stock price data, technical indicators, fundamental factors, and financial news is the first step in a well-documented workflow process (Balaji, 2024). Cleaning, scaling, and feature selection are part of the data preparation step, which occurs before the data is divided into training, validation, and test sets. The machine learning method enhances model performance by leveraging past market behavior after training neural networks, support vector machines, or random forests. The optimal model is chosen for deployment based on its accuracy and operational dependability, following validation and parameter tuning (Alvi et al., 2024). To assist traders and investors in making informed decisions, the algorithm not only tracks market trends but also estimates future stock prices. To increase prediction accuracy, improvement strategies in more complex systems use reinforcement learning, deep learning, and sentiment analysis (Botunac et al., 2024).

One of the primary types of investments is stock market investing; the suggested technique helps users choose the best stock to purchase by identifying patterns and analyzing previous price movements (Hu et al., 2024). By tracking the companies in which users have invested, the suggested approach enables users to create a portfolio that provides a comprehensive view of their entire investment position (Sultana et al., 2024). After applying the regression algorithm to the dataset gathered from the Quandl open-source library for financial and alternative data, the market trend is predicted using a variety of additional indicators, including the MACD, RSI, Bollinger bands, and candlestick pattern detection (Joshi, 2025).

The concept of a hybrid architecture is novel, as it combines anomaly-detection algorithms, social media sentiment analysis, and macroeconomic trend predictions into a single decision-supporting framework (Chopra and Sharma, 2021). An integrated system like this may surpass the traditional silo approach by enabling cross-checking across distinct prediction sources to reduce false positives and improve prediction resilience (Khattak et al., 2023).

**Figure 2:**
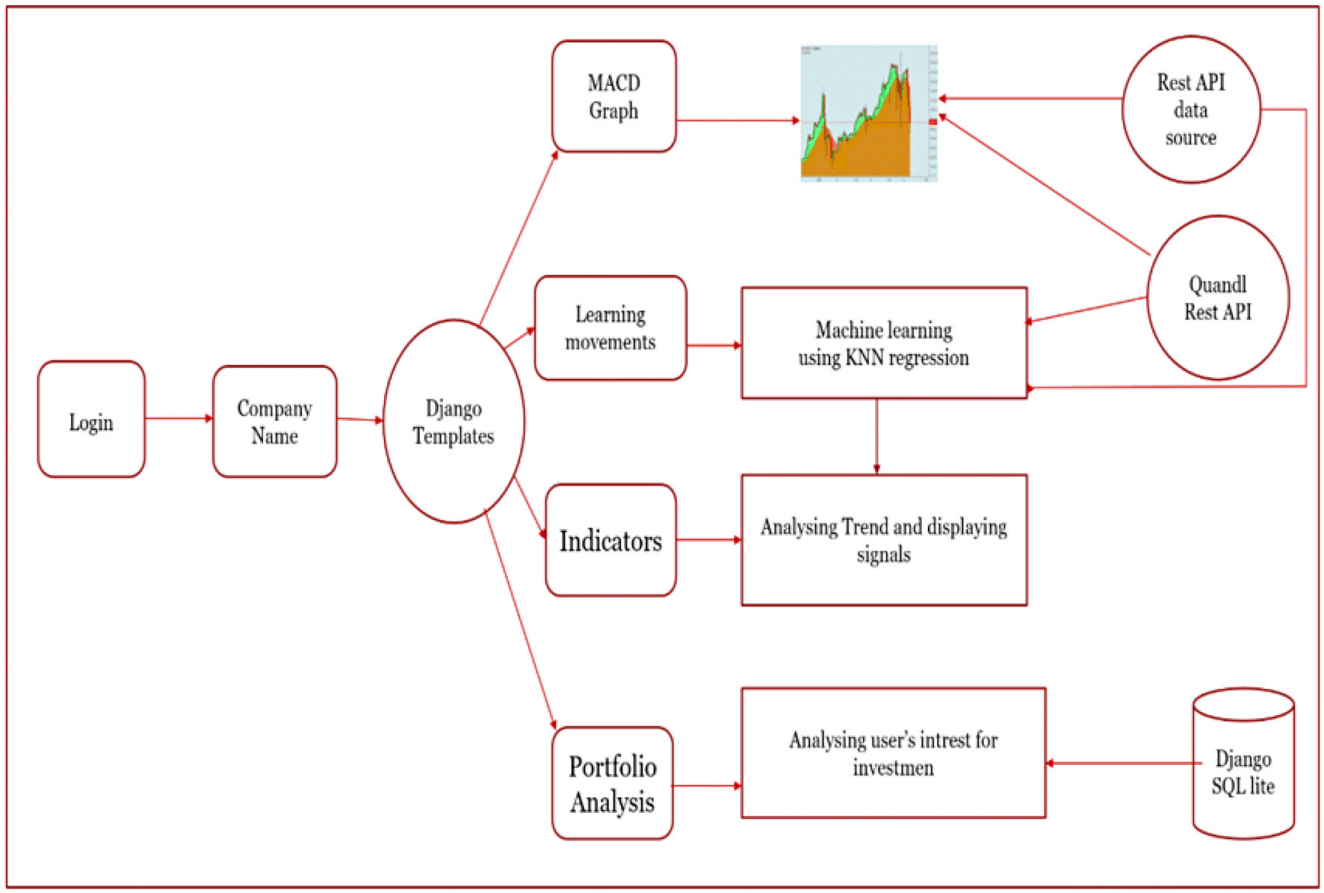
Overview of stock market forecasting (Ananthi and Vijayakumar, 2020).

An overview of the suggested stock market prediction analysis method is shown in Figure 2. Django templates fetch the firm information, the MACD graph measures and displays readings, and portfolio analysis tracks and analyzes stock values and price fluctuations. The k-Nearest Neighbors (kNN) regression machine learning technique is employed to examine stock values further. Additionally, the stock market forecast is analyzed by considering past stock performance and user investment interest, both gathered from SQLite. Lastly, the QuandlRest API is used to produce and display signals (Sarisa et al., 2024; Liu and Lai, 2024; Raheem and Dhannoon, 2023).

### 2.4 Prediction based on fundamental analysis

Financial market predictions based on fundamental analysis involve assessing the intrinsic value of assets by examining industry trends, economic indicators, and corporate financial statements (Dong et al., 2024). In addition to microeconomic elements such as firm profitability, balance sheets, and managerial effectiveness, this approach also relies on macroeconomic factors, including GDP growth, inflation, interest rates, and fiscal policies (Yañez et al., 2024). The Price-to-Earnings Ratio (P/E), Discounted Cash Flow (DCF) analysis, and dividend discount models are among the valuation approaches investors use to determine whether companies are overpriced or undervalued (D'Acunto et al., 2024). Although fundamental research offers a long-term outlook and helps identify growth prospects, it may not be helpful for short-term market fluctuations driven by external shocks and investor sentiment (Lussange et al., 2024). To enhance accuracy and efficiency, fundamental analysis-based financial market predictions are increasingly integrated with cutting-edge technologies, including artificial intelligence (AI) and big data analytics. Modern methods utilize machine learning algorithms to handle large datasets, identify hidden patterns, and improve forecasting capabilities (Das et al., 2024). Traditional fundamental analysis depended on the manual review of financial statements and economic indicators (Rida et al., 2024). Natural language processing (NLP)-based sentiment analysis provides deeper insights by enabling real-time evaluation of market sentiment and financial news. By combining state-of-the-art computational methods with conventional financial concepts, market forecasting is improved, becoming more data-driven and flexible in response to shifting market conditions (Yaqoob and Maqsood, 2024). The dynamic weighting of lead indicators based on the state of the economy is the novelty in this instance. Fundamental analysis becomes more contextual and up-to-date by avoiding the imposition of fixed significance on metrics such as *P*/*E* ratios, dividend yields, and company earnings growth rates. Instead, it employs a dynamic scoring tool that adjusts indicator attention to shifts in sector heritage, geopolitical events, and liquidity trends (Lin and Marques, 2024; Al-Khasawneh et al., 2024). Table 1 provides a concise summary of the key elements, procedures, and challenges associated with financial market prediction based on fun‘damental analysis.

**Table 1:** Fundamental analysis-based prediction of the financial market.

**Classification**	**Explanation**	**Ref**.
Macroeconomic factors	Includes GDP growth, inflation, interest rates, employment data, and fiscal/monetary policies affecting market trends.	Agrawal et al., 2024
Financial ratios	Key valuation metrics such as Price-to-Earnings (*P*/*E*) ratio, Price-to-Book (*P*/*B*) ratio, Dividend Yield, and Return on Equity (ROE).	Salman et al., 2024
Microeconomic factors	Focuses on individual company performance, including revenue, earnings, assets, liabilities, and management efficiency.	Huang et al., 2023a,b
Industry and sector analysis	Examines industry trends, competitive advantages, market demand, and regulatory impacts on specific sectors.	Li et al., 2023
Market sentiment and news	It uses sentiment analysis and NLP to assess the impact of financial news, earnings reports, and investor sentiment on stock prices.	Manogna and Anand, 2023
Predictive models	Incorporates AI, machine learning, and statistical models to analyze fundamental data for more accurate predictions.	Nie et al., 2024
Challenges and limitations	It may not be effective for short-term trading, as speculation, external shocks, or behavioral biases can significantly influence market prices.	Fischer et al., 2024

## 3 Machine learning techniques in financial market prediction

Data-driven algorithms that employ machine learning techniques are used in financial market prediction to analyze past market data, identify trends, and make predictions. To predict stock prices and classify market patterns, supervised learning techniques like regression and classification models are employed (Sangeetha and Alfia, 2023). Unusual trade patterns and hidden market structures can be identified using unsupervised learning techniques, such as clustering and anomaly detection. Time-series forecasting often employs deep learning models, particularly recurrent neural networks (RNNs) and long short-term memory (LSTM) networks (Wu et al., 2024). Adaptive trading methods are enabled by reinforcement learning, which is likewise becoming increasingly popular. Machine learning improves automated trading systems, risk assessment, and forecast accuracy by combining AI with technical and fundamental analysis (Zhang et al., 2023).

### 3.1 Financial markets and blockchain technology

Blockchain technology is reshaping financial markets by improving the integrity, transparency, and auditability of transaction data (Bustos and Pomares-Quimbaya, 2020; Phuoc et al., 2024; Ayyildiz and Iskenderoglu, 2024). Transactions recorded on distributed ledgers create a time-stamped, tamper-resistant trail that reduces the scope for fraud and misreporting, simplifies post-trade reconciliation, and supports regulatory oversight (Bustos and Pomares-Quimbaya, 2020; Phuoc et al., 2024). Tokenization further enables traditional assets, such as equities or funds, to be represented as digital tokens, thereby increasing market accessibility through fractional ownership and 24/7 trading (Ayyildiz and Iskenderoglu, 2024).

For financial market prediction, the key contribution of blockchain is the quality and granularity of data it generates. On-chain transaction flows, wallet interactions, and token transfers provide real-time signals about liquidity shifts, market concentration, and systemic risk (Rath et al., 2023; Islam et al., 2024; Oyewola et al., 2023). Machine learning models can combine these transparent, high-frequency data streams with conventional market indicators to detect abnormal behavior, anticipate liquidity crises, and improve risk scoring and anti-money-laundering analytics (Campisi et al., 2023; Ahmed et al., 2022).

### 3.2 Possible effect on forecasting the market

Market forecasting has been significantly impacted by the integration of cutting-edge technologies, including blockchain, artificial intelligence, and machine learning, which enhance precision, speed, and efficiency. To identify hidden patterns and trends that traditional models may overlook, machine learning algorithms analyze vast datasets (Zheng H. et al., 2024). Enhancing data security and transparency through blockchain technology reduces fraud and fosters greater confidence in financial transactions. Furthermore, sentiment research from social media and news sources offers real-time market data, enabling more flexible forecasting (Shaban et al., 2023). Market forecasting remains a dynamic issue despite technological breakthroughs, as external factors such as economic policy, geopolitical events, and market psychology continue to introduce uncertainty (Huang et al., 2024).

Enhancements in processing speed, predictive accuracy, and the capacity to manage vast datasets with previously unattainable efficiency are just a few of the ways that quantum computing may impact market prediction (Ghosh et al., 2024). Financial forecasting may change as a result of quantum algorithms, particularly in portfolio optimization, which enable quicker and more thorough examinations of market scenarios and enhance the flexibility of investment plans (Wei et al., 2024). By resolving problems such as overfitting in conventional machine learning models and enhancing parameter tuning, the exponential processing capacity of quantum computing enhances prediction accuracy (Lee et al., 2024). It is also ideal for analyzing large datasets to identify subtle trends due to its ability to process enormous volumes of data simultaneously. The quantum machine Learning that leverages quantum mechanics holds even more potential, as it can accelerate model training and improve stock price forecasts by reducing complexity and enhancing convergence (Mamun et al., 2024). However, incorporating quantum computing into market prediction is challenging due to existing limitations on qubit count, coherence durations, and error rates, as well as the requirement for specialized knowledge (Zheng J. et al., 2024). Instead of relying on past patterns, its uniqueness lies in using ensemble machine learning models that continuously adjust their weightings when marketing conditions unexpectedly shift, such as in politically charged or rapidly evolving markets. Forecasts become less vulnerable to uncertainty when an adaptive recalibration process is used to detect emerging patterns that static models often miss (Hajj and Hammoud, 2023; Mahalakshmi et al., 2021). The various elements that may affect market forecasting and their potential impacts on both short-term and long-term market behavior are outlined in Table 2.

**Table 2:** Market forecasting influential factors and their possible impact.

**Factor**	**Effect on market forecasting**	**Impact type**	**Examples**	**Ref**.
Economic indicators	Key indicators, such as GDP growth, inflation, and unemployment rates, can provide a clear view of the market's future performance.	Predictive/deterministic	GDP growth rates, unemployment reports, and inflation trends.	Billah et al., 2024
Technological advancements	New technologies can disrupt or boost specific markets, altering trends.	Long-term growth/disruption	AI, renewable energy, and biotech developments.	Wu, 2024
Consumer sentiment	Confidence or pessimism in consumer spending habits can shape demand and supply predictions.	Short-term volatility/trend	Consumer surveys, retail sales data, and confidence indices.	Memiş et al., 2024
Market trends	Existing trends and cycles can impact both short-term and long-term market movements.	Cyclical/trend-based	Bull markets, bear markets, sector rotations.	Hamadou et al., 2023
Supply chain disruptions	Problems in supply chains can lead to price increases, delays, and shifts in the market.	Short-term volatility	Shipping delays, material shortages, trade restrictions.	Qiu et al., 2023
Currency fluctuations	Changes in currency exchange rates can impact international trade and investment flows.	Volatility/international	USD/EUR exchange rate shifts and emerging-market currencies.	Beniwal et al., 2023

### 3.3 Financial modeling and quantum computing

The process of developing mathematical representations of a business, investment, or financial instrument's financial performance is known as financial modeling. It entails forecasting future financial results and supporting decision-making using data, statistical techniques, and algorithms (Huang et al., 2023b). To address complex financial issues such as asset allocation, risk management, portfolio optimization, and option pricing, traditional financial modeling approaches rely on classical computers (Corizzo and Rosen, 2023). Conversely, quantum computing leverages the principles of quantum physics to process data in fundamentally distinct ways from traditional computers. By leveraging quantum superposition and entanglement, quantum computers can process large volumes of data and perform intricate computations significantly faster than conventional computers (D'Uggento et al., 2025). Despite its significant theoretical promise, quantum computing currently faces substantial technical limitations that restrict its applicability to financial forecasting (Patil et al., 2022). Qubit decoherence, noise, limited qubit counts, and low gate fidelity prevent the reliable scaling of algorithms such as the Quantum Approximate Optimization Algorithm (QAOA) and the Variational Quantum Eigensolver (VQE) (Song et al., 2024). Many reported advances rely on simulations rather than actual quantum hardware, and as of 2024, no verified large-scale financial implementation of quantum forecasting exists (Mishra et al., 2024). Therefore, quantum-assisted models should be regarded as exploratory research tools rather than practical forecasting solutions, with real-world deployment still requiring major technological breakthroughs (Zou et al., 2023).

Potential developments in portfolio optimization pose a challenging problem in investment management: minimizing risk while maximizing returns, particularly as the number of assets increases. The exponential growth in the number of potential asset combinations makes classical techniques, such as Markowitz's mean-variance optimization, computationally intensive (Zhang and Chen, 2023). By leveraging superposition states of qubits to compute multiple solutions simultaneously, quantum algorithms, particularly the Quantum Approximate Optimization Algorithm (QAOA), can overcome this difficulty and reduce the time required to identify the optimal portfolio (Jha et al., 2025a). QAOA is highly effective for large-scale portfolio optimization because it uses quantum circuits to explore the solution space and iteratively updates the quantum state to minimize a cost function that represents the portfolio's risk-adjusted return (Wang et al., 2024a). The combination of traditional machine learning models with the high-dimensional optimization toolbox of quantum computing in market prediction offers a novel advantage. Financial models can now process vast amounts of multidimensional data, such as worldwide market correlations, at previously unheard-of rates thanks to this integration, which may help them identify non-linear and arbitrage linkages that were previously impossible to calculate (Bansal et al., 2022).

**Figure 3:**
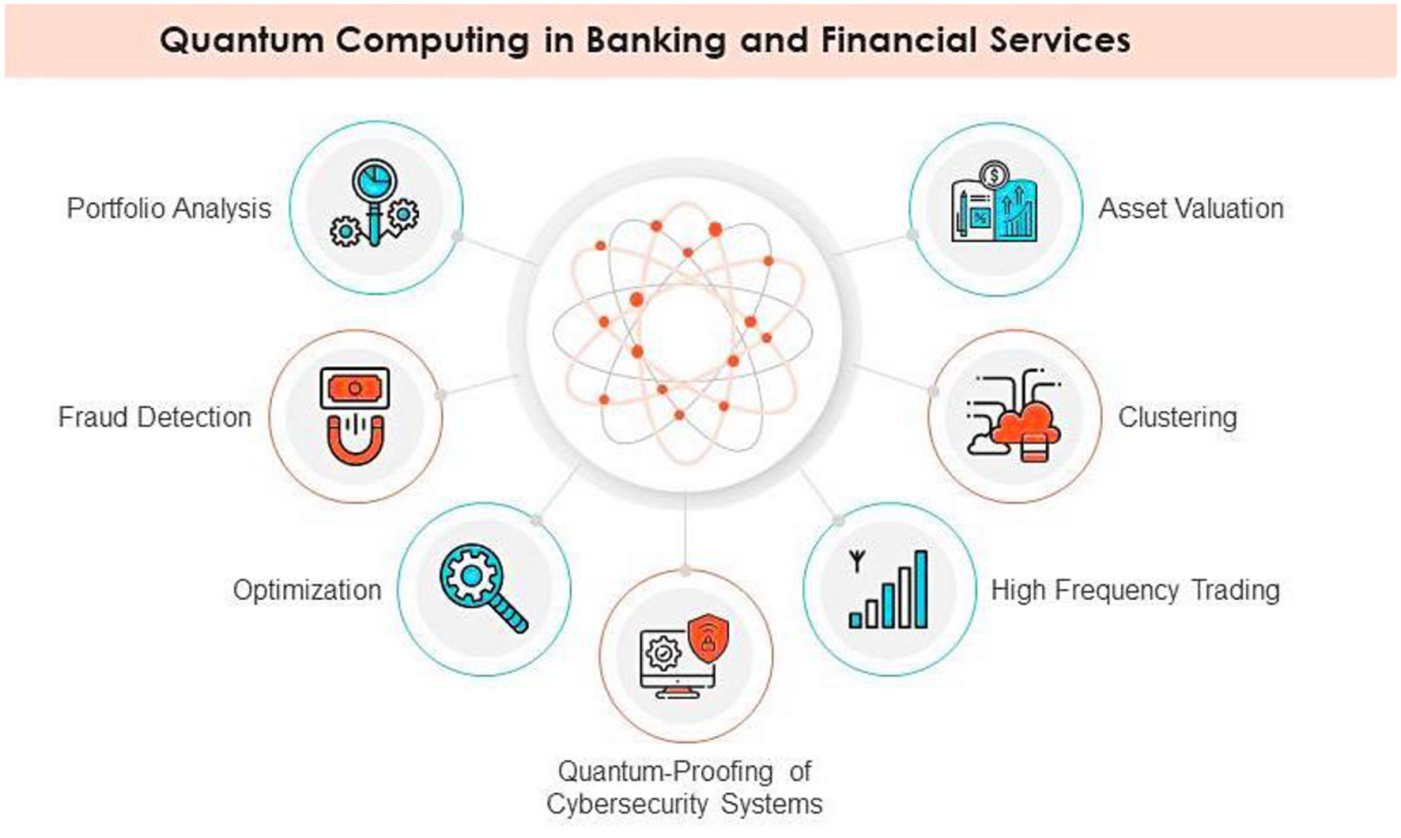
Financial services and banking using quantum computing.

Key uses of quantum computing in the finance industry are depicted in Figure 3. The fundamental technology is represented by a quantum-inspired image at the center, with seven surrounding icons showing its applications. These include high-frequency trading for faster algorithmic execution, asset valuation for more precise pricing models, clustering for data segmentation, portfolio analysis for investment optimization, fraud detection to identify suspicious transactions, and quantum-resistant cybersecurity for enhanced data protection. The graphic illustrates how leveraging quantum computing to improve efficiency, security, and decision-making processes could revolutionize banking and finance.

### 3.4 Data collection and purification

First, compile information from Twitter. This social networking site was chosen because it is succinct. Sentiment140, a state-of-the-art dataset, was utilized in conjunction with tweet data directly obtained from Twitter. Once the data has been gathered, use a reduction technique to eliminate any spam, redundant, pointless, or unnecessary tweets (Zheng S. et al., 2024). Preprocessing involves applying ML techniques to extract features and identify sentiment from the cleaned dataset. Through this procedure, the unprocessed Twitter data were transformed into a standard dataset that included tweets with their projected sentiments, represented as positive, negative, or neutral (1, −1, or 0), along with a feature set.

**Figure 4:**
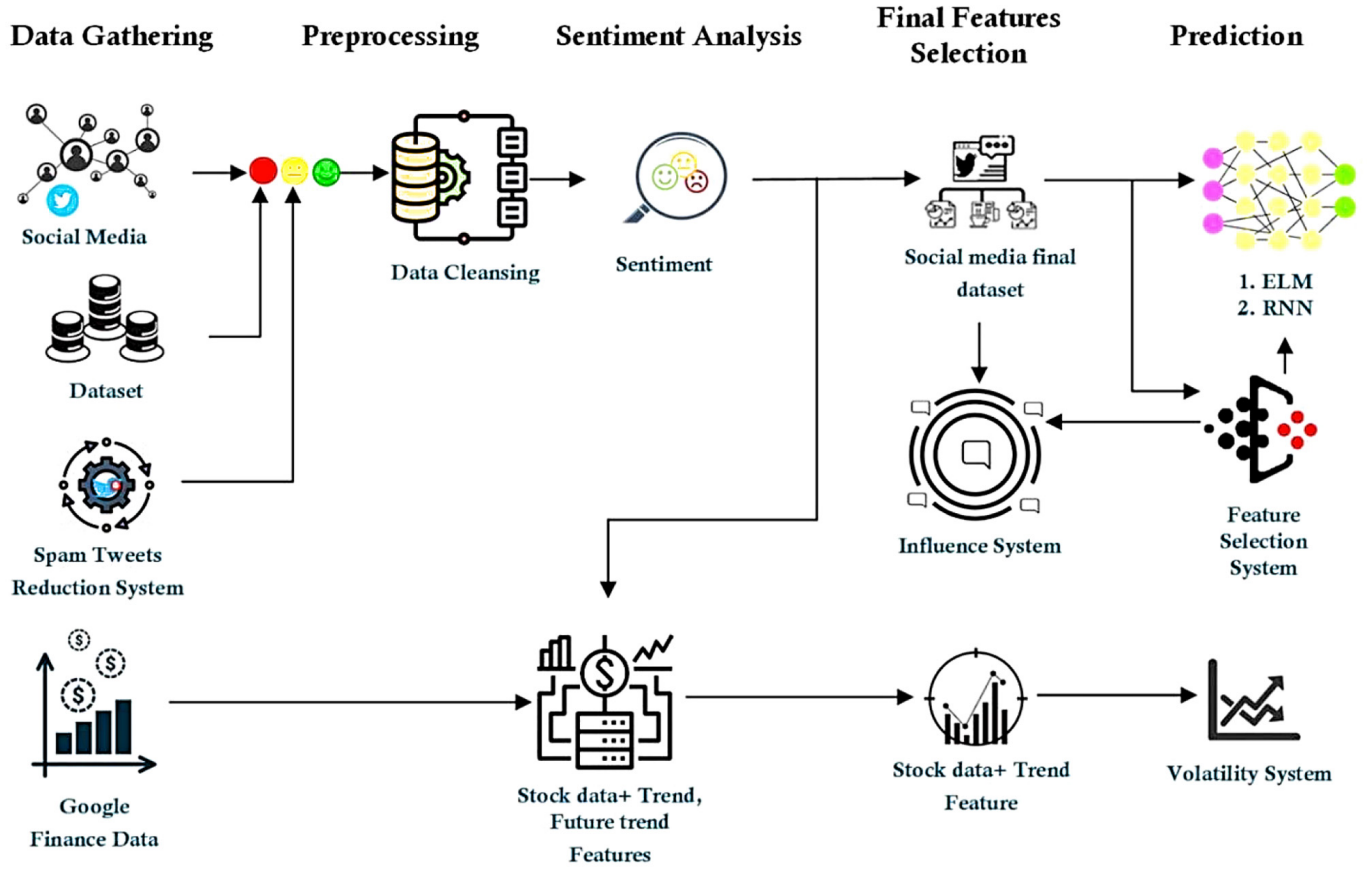
Flow diagram of suggested approach (Albahli et al., 2022).

Additionally, neutral tweets may lead to an imbalance throughout the training phase, which might impair the classifier's effectiveness (Singh and Malhotra, 2024; Ju and Zhu, 2024; Ritu et al., 2025; Mazinani et al., 2024). We employed a straightforward technique to eliminate neutral tweets from the dataset by identifying them based on their label (i.e., 0) and filtering them out, resulting in a smaller dataset devoid of neutral tweets (Tekouabou et al., 2023). Neutral tweets are eliminated from the dataset as they have no bearing on the prediction process. Neutral tweets must be eliminated for two reasons: (i) they do not contribute significantly to opinion mining because they do not contain any sentiment or opinion polarity, and (ii) adding a neutral set of tweets results in a larger dataset, which adds needless overhead for the classifier during model training (Boussatta et al., 2025). Figure 4 shows the total architecture.

Second, utilize Google Finance's stock market data, which includes global historical stock data. The price data for selected stocks is downloaded from the service provider in a CSV file. The collected data include seven features: date, open, high, low, close, volume, and adjusted close (Abdelfattah et al., 2024). These features indicate the trade date, opening price, highest and lowest trading prices, closing price, traded shares, and the stock's closing price when investors receive their dividends, respectively (Tang et al., 2024). Additionally, this data is preprocessed by deleting some columns, such as adjusted close price, volume, and opening price, and adding some computed values based on existing characteristics [such as 5-day price difference, 10-day price difference, extrapolation prices during vacations, and return of the market (RM)] (Abdou et al., 2024). The following justifies the inclusion of the computed values: The 5- and 10-day price difference gives a quick overview of the stock's historical performance. To complete the dataset's chronology, weekend closing prices have been projected, which may enhance the model's overall accuracy. To provide an investor with a probabilistic understanding of risk vs. expected reward, the market return (RM) is calculated (Che W. et al., 2024). Following preprocessing for both data sources, the next step is to train the model and make stock predictions. Using the features extracted from the Twitter and Google Finance datasets, an Extreme Learning Machine (ELM) and RNN-based model has been trained, with the first 70% of the datasets allocated for training and the remaining 30% for testing and validation. The results and discussion section provides more information about the datasets incorporated (Akşehir and Kiliç, 2024). In this context, the invention is an AI-driven automated pipeline for cleaning market data that leverages deep anomaly-detection networks to identify and correct discrepancies across diverse datasets. By identifying the statistical signature of reliable financial data, the method goes beyond conventional preprocessing by training prediction models using only high-fidelity, bias-reduced inputs (Awad et al., 2023).

### 3.5 Using reinforcement learning (RL) to predict financial markets

Through market experimentation with reinforcement learning (RL), agents can develop the most effective trading strategies. Because RL employs reward-based decision-making that operates effectively under ambiguous market conditions and does not rely on predetermined labels, it is in opposition to supervised learning (Dixit and Soni, 2023). RL models, which include Deep Q-Networks (DQN), Policy Gradient Methods, and Proximal Policy Optimization (PPO), enable real-time market adjustments to trading strategies and the identification of patterns from past market data (Ahmed et al., 2024). The framework helps investment managers with risk assessments and trading tasks, allowing traders to maximize profits while minimizing risk exposure. RL-based models are better for financial decision systems because they can continually adjust to market movements, which gives automated high-frequency trading techniques a deployment edge. It is essential to address issues of overfitting, computational complexity, and erratic market behavior to use RL (Kanaparthi, 2024a) successfully. Deep reinforcement learning (DRL), multi-agent reinforcement learning (MARL), and meta-learning are the primary focuses of recent advancements in reinforcement learning (RL) for financial market prediction, which enhance trading tactics and market flexibility, by fusing reinforcement learning (RL) with deep neural networks, DRL enables models such as Transformers-based RL and attention mechanisms to recognize intricate market trends (Cao et al., 2024). To improve strategy resilience in very turbulent markets, Multi-agent RL (MARL) integrates cooperative and competitive agents that mimic real-world trade dynamics.

Furthermore, by learning from a variety of datasets, meta-learning RL shortens retraining times. It enhances decision-making under uncertainty, enabling models to swiftly adjust to shifting market regimes (Ting et al., 2024). Quantum-enhanced RL is another breakthrough that leverages quantum computing to accelerate the handling of financial optimization problems. These developments elevate RL above conventional algorithmic trading, increasing its scalability, adaptability, and effectiveness for risk assessment, portfolio management, and high-frequency trading in actual financial markets (He et al., 2024). Multi-agent reinforcement learning is a novel approach in which individual trading agents with different bias strategies cooperate and compete in a fictitious market environment. In addition to teaching agents how to optimize returns, this kind of setup exposes the collective model to a broader range of market behaviors, thereby improving its ability to forecast real-world market dynamics and adapt to trends (Pokhrel et al., 2022).

**Figure 5:**
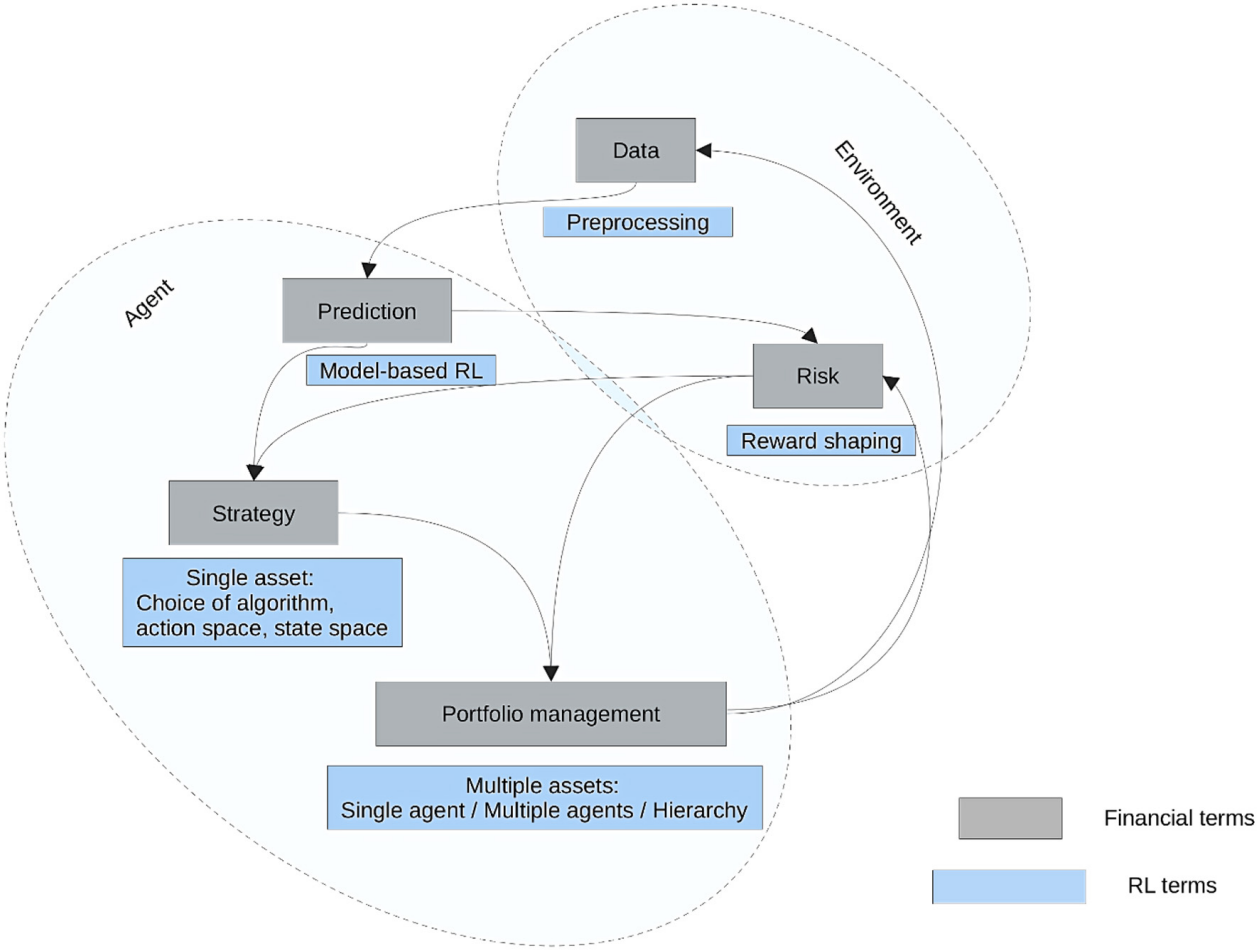
An outline of the key financial ideas covered and their real-world equivalents (Millea, 2021).

A framework for financial market prediction and portfolio management utilizing reinforcement learning (RL) is illustrated in Figure 5, highlighting the connections between RL and economic concepts. The two main parts of the figure are the Agent and the Environment. Risk management and data pretreatment are included in the Environment; reward shaping affects RL-based risk assessment, while preprocessing improves input data (Bodislav et al., 2024). Model-based RL facilitates forecasting, and various RL methods can be applied to one or more assets using either single-agent or multi-agent techniques. The Agent comprises prediction, strategy, and portfolio management. By graphically distinguishing between RL-specific terminology (blue boxes) and financial terms (gray boxes), the diagram highlights the integration of RL approaches into financial decision-making (Habbab and Kampouridis, 2023). This methodical approach demonstrates how RL can enhance financial forecasts, mitigate risks, and optimize asset allocation through ongoing learning and adaptation (Biju and Thomas, 2023).

### 3.6 Comparative analysis and critical evaluation of ML technique

While the aforementioned ML techniques offer significant promise, a critical evaluation of their strengths, weaknesses, and suitability is essential for practical application. Table 3 provides a comparative summary of key models. Deep learning models, particularly LSTMs and Transformers, excel at capturing complex temporal dependencies and non-linear patterns in vast datasets, often achieving state-of-the-art prediction accuracy (Zhang et al., 2023). However, their primary weaknesses are their “black-box” nature, high computational cost, and propensity to overfit noisy financial data, requiring extensive regularization and validation (Martin and Nagel, 2021; Farzaneh et al., 2021; Theng and Bhoyar, 2023; Ahlstrom et al., 2020; Toma et al., 2020; Kumar et al., 2020). In contrast, traditional models like SVMs and Random Forests offer greater interpretability and robustness to overfitting but may struggle to capture long-term sequential dependencies and require meticulous feature engineering (Kumbure et al., 2022). Reinforcement Learning (RL) and Deep RL represent a paradigm shift by optimizing for cumulative reward (e.g., profit), allowing for dynamic strategy adaptation (Cao et al., 2024). Their primary challenges lie in the high variance in learning, sensitivity to reward function design, and the immense computational cost of simulating realistic market environments for training (Millea, 2021).

**Table 3:** Comparative analysis of selected machine learning models for stock prediction.

**Model category**	**Example techniques**	**Key strengths**	**Major weaknesses and challenges**	Typical performance metrics (range)^*^	**Key references**
Traditional ML	SVM, Random Forest, XGBoost	High interpretability, robust to overfitting, efficient with structured features.	Limited capacity for raw sequence data requires extensive feature engineering.	Accuracy: 55–70%; RMSE: Varies by asset	Kurani et al., 2021; Agrawal et al., 2024; Kumbure et al., 2022
Deep learning (sequential)	LSTM, GRU, Temporal CNN	Captures long-term temporal dependencies, learns features automatically from raw data.	Black-box nature, computationally intensive, prone to overfitting on noise.	Accuracy: 60–75%; RMSE: Often lower than trad. ML	Bhandari et al., 2022; Zhang et al., 2023; Rezaei et al., 2020; Lu et al., 2020
Deep learning (attention-based)	Transformers, Bidirectional Encoder Representations from Transformers (BERT)-fintech	Models long-range dependencies powerfully, excels with heterogeneous data (text + series).	Very high computational cost, massive data requirements, and interpretability challenges.	Accuracy: 65–78% (on sentiment-aided tasks)	Muhammad et al., 2023; Yañez et al., 2024; Wang et al., 2022
Reinforcement learning	DQN, PPO, Multi-Agent RL	Optimizes for profit/risk directly, enabling adaptive trading strategies.	Extremely high training variance, complex reward shaping, and sim-to-real gap.	Sharpe Ratio, Maximum Drawdown (Backtested)	Qiu et al., 2023; Dixit and Soni, 2023; He et al., 2024; Millea, 2021
Hybrid/ensemble models	LSTM+CNN, VMD+XGBoost, Stacking	Mitigates individual model weaknesses, often achieves peak accuracy.	Increased system complexity, compounded by interpretability issues.	Accuracy: Can exceed 70%; Lower RMSE	Botunac et al., 2024; Zhang and Chen, 2023; Sonkavde et al., 2023; Shah et al., 2022

Hybrid models attempt to mitigate individual model weaknesses by combining architectures, such as integrating LSTM's memory with CNN's feature extraction or blending model outputs via ensemble methods (Botunac et al., 2024). While these often outperform standalone models, they increase system complexity and can become even less interpretable. A critical unresolved challenge across all methods is model robustness under regime shifts—market behavior changes due to economic crises, regulatory shifts, or geopolitical events can swiftly degrade a model trained on historical data (Sarisa et al., 2024). Furthermore, the signal-to-noise ratio in financial data is exceptionally low, meaning even highly accurate models may have limited economic utility after accounting for transaction costs and slippage (Martin and Nagel, 2021). Future research must pivot toward developing more adaptive, explainable, and economically grounded models that can self-diagnose performance decay and incorporate real-time structural break detection.

These findings, as shown in Table 4, are that deep learning and hybrid architectures consistently outperform traditional ML methods across different markets. However, performance varies by dataset characteristics, market volatility, and the inclusion of sentiment or external features.

**Table 4:** Comparative performance of ai models for stock market prediction.

**Model**	**Dataset**	**Metric**	**Performance**	**Source**
LSTM	S&P 500	RMSE	0.028	Nabipour et al., 2020
Bi-LSTM	NASDAQ	MAE	0.021	Hamayel and Owda, 2021
Transformer	DSE (Bangladesh)	Accuracy	92.4%	Muhammad et al., 2023
Random forest	NIFTY 50	RMSE	0.041	Sonkavde et al., 2023
Hybrid CNN-LSTM	Shanghai Index	MAE	0.018	Rezaei et al., 2020
DQN reinforcement agent	S&P 500	Sharpe Ratio	1.47	Ayitey Junior et al., 2023

## 4 Applications of machine learning in stock price prediction

Predicting stock prices has been revolutionized by machine learning (ML), enabling the creation of automated, data-driven, and highly accurate forecasting models. To forecast future trends, supervised learning methods such as support vector machines (SVM), deep neural networks, and linear regression utilize past stock prices, trade volumes, and macroeconomic variables (Jiang, 2021). Unsupervised learning techniques, such as anomaly detection and grouping, reveal hidden investor and market trends. NLP analyzes financial news and social media sentiment to forecast market movements, while RL continually optimizes trading techniques by learning from market conditions (Chhajer et al., 2021). Furthermore, hybrid models integrate many machine learning approaches to produce more reliable predictions, enhancing risk management and portfolio optimization. In turbulent markets, these apps enhance high-frequency trading strategies, mitigate risk, and help investors make more informed decisions (Hu et al., 2021).

### 4.1 Using technical analysis to predict stock prices

Technical analysis predicts future stock price movements by utilizing statistical indicators, trade volume, and previous price data. By automating pattern detection, reducing human bias, and enhancing predicted accuracy, machine learning enhances technical analysis (Rezaei et al., 2020). To determine buy and sell signals, algorithms such as SVM, decision trees, deep learning, and reinforcement learning examine price trends, moving averages, relative strength index (RSI), and candlestick patterns. To improve forecasts, sophisticated models utilize technical indicators such as Fibonacci retracements, Bollinger Bands, and MACD (Moving Average Convergence/Divergence) (Rouf et al., 2021). High-frequency trading (HFT), algorithmic trading, and risk management all extensively leverage machine-learning-driven technical analysis, enabling traders to make informed decisions in volatile markets. However, because market conditions can occasionally defy solely technical indications, it must be used in conjunction with risk assessment procedures (Carta et al., 2020). New developments in technical analysis improve stock price prediction by utilizing deep learning, AI-driven models, and hybrid approaches. By learning optimal buy-sell positions in real time and dynamically adjusting to market conditions, Deep Reinforcement Learning (DRL) has revolutionized trading techniques (Zhang and Lou, 2020). Time-Series Transformers and finance BERT are two examples of transformer-based models that enhance pattern detection in intricate stock data.

Furthermore, to improve transparency and assist traders in understanding why particular signals yield specific forecasts, Explainable AI (XAI) approaches have been incorporated (Wang et al., 2022). Large financial datasets can now be processed more quickly and effectively thanks to advancements in quantum computing for technical analysis (Ahmed et al., 2022). Additionally, hybrid models that incorporate technical indicators and sentiment analysis provide more comprehensive market insights, reducing dependence on price changes alone. These innovations significantly increase the precision, flexibility, and resilience of stock price forecasts in volatile market conditions (Lu et al., 2020). Predictive accuracy is improved by merging machine learning algorithms with conventional chart-based techniques and indicator-based forecasts. More advanced models not only use static patterns from previous occurrences, such as historical trends, but also dynamically adjust technical indicators, such as moving averages, RSI, and Bollinger Bands, in response to changing market conditions. The technological simplification of technical analysis via real-time data inputs, reduced human bias, and improved responsiveness to erratic market fluctuations makes it revolutionary (Bhandari et al., 2022; Cernevičiene and Kabašinskas, 2024).

**Figure 6:**
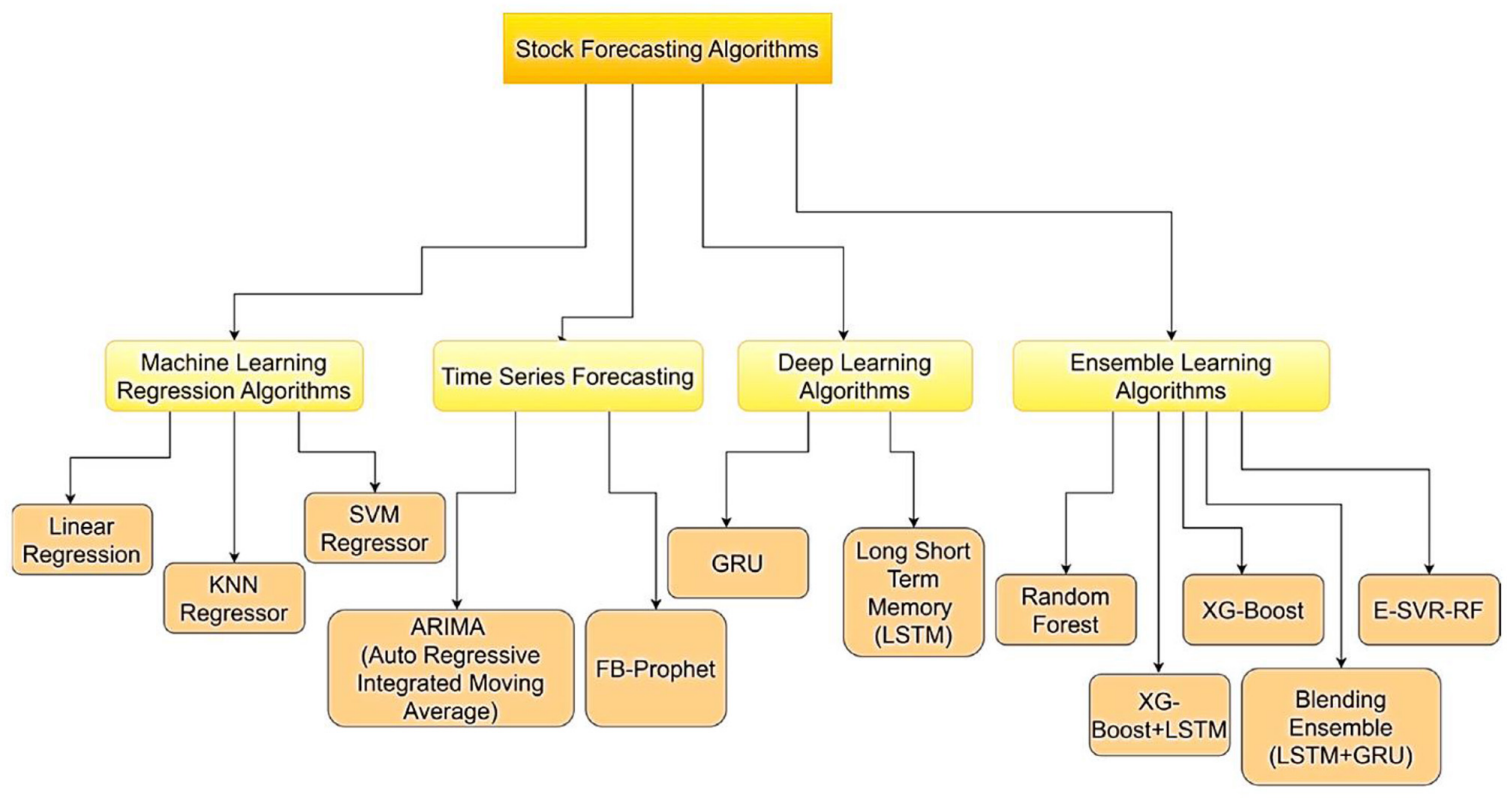
An algorithm for stock forecasting (Sonkavde et al., 2023).

A hierarchical overview of stock forecasting algorithms is presented in Figure 6, which categorizes them into four primary groups: ensemble learning, deep learning, time series forecasting, and machine learning regression. Traditional models that forecast stock values based on patterns in past data, such as Linear Regression, KNN Regressor, and SVM Regressor, are included in the Machine Learning Regression Algorithms section. Analysis of sequential market data is the primary focus of time series forecasting techniques, such as FB-Prophet and ARIMA. GRU and LSTM, which capture intricate relationships between stock prices over time, are highlighted in the Deep Learning Algorithms area. Lastly, by integrating several approaches, Ensemble Learning Algorithms combine many models, such as Random Forest, XG-Boost, E-SVR-RF, and hybrid models like XG-Boost+LSTM and Blending Ensemble (LSTM+GRU), to increase prediction accuracy.

### 4.2 Analysis of sentiment

Sentiment analysis has gained significant importance over the past decade, mainly due to the vast amount of textual data available on news and social media platforms. It is possible to mine this textual data to find user opinions for many application areas (Wu et al., 2021a). Data mining and machine learning are essential for analyzing the sentiment of this vast amount of textual data. As a result, academics studying machine learning have investigated methods for extracting people's opinions from these sites (Jing et al., 2021). Depending on what they include, tweets may be divided into many kinds. Yuan (2016) investigated sentiment classification techniques based on rules, vocabulary, and machine learning (Khedr et al., 2021).

Regarding the methods based on the lexicon, feature scoring, and word Count methods were attempted. Naïve Bayes (NB), maximum entropy (ME), and support vector machines (SVM) were employed in the machine learning-based approach (Urolagin et al., 2021). Bag of Words and Part-of-Speech Linguistic Annotations (BoW) were compared with N-Gram characteristics. They found that BoW was a straightforward, practical feature that yielded the best results (Swathi et al., 2022). Additionally, the language aspects performed better. NB was used to conduct a poll on Twitter data classification. After examining the tweets, they concluded that the data is highly organized and diverse, and can be categorized as either good, neutral, or negative (Gülmez, 2023). It is possible to perform sentiment analysis of user opinions across various application areas. To categorize movie review data from Twitter using unigram features, bigram features, and a combination of unigram and bigram features, Joshi and Tekchandani (2016) conducted a comparative examination of SVM, ME, and NB machine learning algorithms (Kurani et al., 2021). They discovered that SVM outperformed the other classifiers. Examined logistic regression (LR) and neural networks using two weighting systems on tweets on technological stocks, such as Facebook, Google, Twitter, and Tesla: unigram term frequency (TF) and bigram TF inverse document frequency (TF-IDF) (Tschora et al., 2022). They deduced from the experimental findings that the classifiers produced identical total accuracies. Nevertheless, empirical tests revealed that unigram TFIDF performed better than TF (Bhandari et al., 2022). Like social media, news is a significant external force that influences stock markets and disseminates information about important events related to equities. For this reason, machine learning researchers also conducted sentiment analysis on news data (Jiang et al., 2020). After doing a sentiment analysis of the news, Dang and Duong (2016) divided the news into three classes: upward, neutral, and downward. To find a link between stock prices and financial news, they applied SVM to data on stock prices and business news related to the VN30 Index. They discovered that news and stock prices are correlated. (Tirea and Negru 2015) extracted stock-related information from news ontologies that influenced stock behavior using automated text classification (Torres et al., 2020). They discovered a connection between news and stock price movements. They used news about businesses listed on the Bucharest Stock Exchange. Google Custom Search was used to crawl the news data (Tirkolaee et al., 2021). Breaking news is shared on social media sites like Twitter in addition to news websites. Alostad and Davulcu (2017) predicted the hourly direction of the stocks of 30 businesses listed on the DJI using financial news from the NASDAQ website and breaking news from Twitter. They demonstrated that the accuracy of hourly directional predictions for the stocks included in news pieces increased significantly due to the information they carried (Raubitzek and Neubauer, 2021). Since sentiment analysis uses a vast amount of textual data, such as news headlines, analyst coverage, and social media postings, to measure market sentiment, it contributes behavioral input to stock predictions. The novel aspect is the combination of advanced natural language processing (NLP) with an understanding of the context-specific language used in the financial realm. This enables the system to identify subtle tones, such as undertones that sound doubtful or cautiously hopeful. As a result, the model can predict market responses before they fully affect prices (Salahshour et al., 2024; Boozary et al., 2025).

### 4.3 Hybrid stock prediction model employing deep neural networks and prediction rule ensembles

Hybrid stock prediction models have become increasingly prominent because they integrate multiple learning techniques within a single forecasting framework. Deep Neural Networks (DNNs) are highly effective at capturing complex, non-linear relationships in financial variables such as historical prices, trading volume, and macroeconomic indicators, thereby identifying patterns that traditional algorithms often miss (Sharma et al., 2021). Prediction Rule Ensembles (PREs), on the other hand, contribute transparency and robustness by generating rule-based explanations that reduce overfitting and enhance model stability (Sebastião and Godinho, 2021). When combined, PREs and DNNs create a complementary architecture that balances interpretability with predictive strength, improving the overall reliability of stock forecasting systems (Taye, 2023).

Recent advancements in hybrid approaches extend beyond conventional PRE–DNN integration. Transformer-based architectures such as BERT, GPT, and Temporal Fusion Transformers (TFT) have been incorporated into hybrid models to leverage attention mechanisms that focus on the most influential market signals, thereby improving both accuracy and explanatory depth (Chan et al., 2023). Additional developments explore quantum-assisted neural networks, which promise enhanced computational speed for processing large-scale financial data in real time, although these systems are still in early experimental stages (Barrera-Animas et al., 2021). Hybrid systems also increasingly integrate sentiment analysis, in which natural language processing (NLP) extracts investor sentiment from news, reports, and social media streams to refine prediction accuracy and capture the behavioral dimensions of market movement (Barrera-Animas et al., 2021).

**Figure 7:**
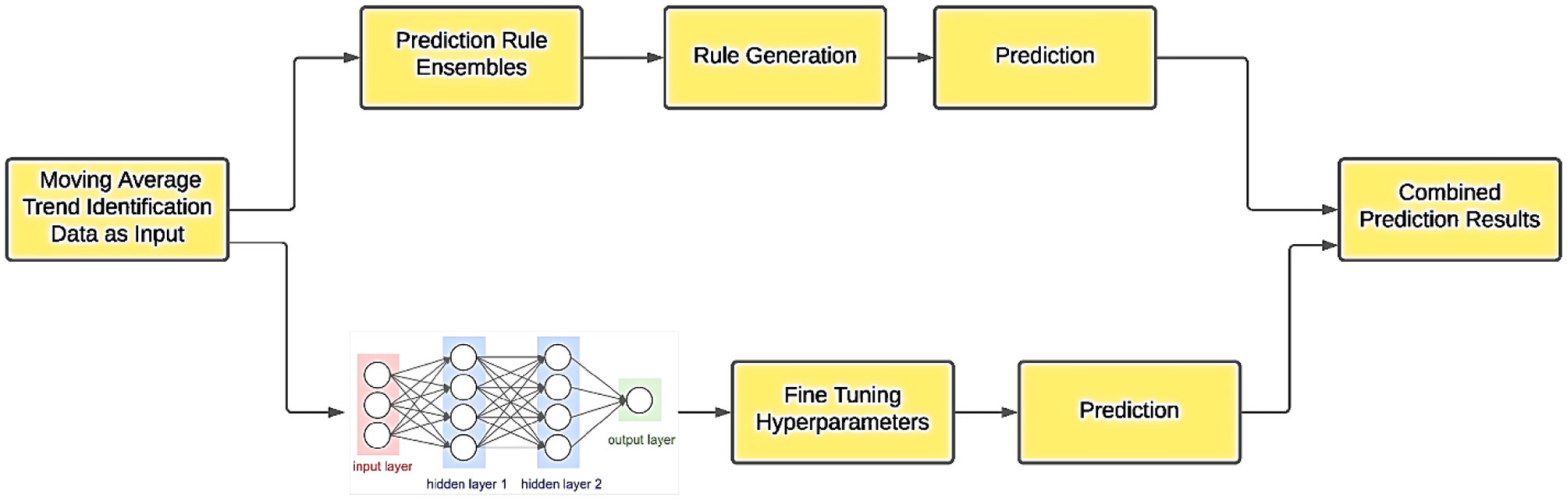
Hybrid model for forecasting stock prices (Srivinay et al., 2022).

A carefully engineered hybrid model addresses important limitations found in standalone deep learning systems, including their tendency to overfit, instability under minor data shifts, and limited interpretability. By combining rule-based reasoning from PREs with the pattern-recognition capabilities of DNNs, the hybrid architecture provides a more stable, adaptable, and transparent forecasting solution that performs well in high-dimensional, rapidly changing market environments (Shah et al., 2022; Biju and Thomas, 2023). This balanced integration enhances risk management and supports more informed decision-making for traders (Figure 7). To overcome instability in traditional hybrid approaches, recent studies propose PRE–DNN frameworks specifically designed to mitigate sensitivity to variable selection and training data fluctuations, thereby further strengthening the reliability of stock prediction outcomes (Shehadeh et al., 2021).

### 4.4 Stock market machine learning system

**Figure 8:**
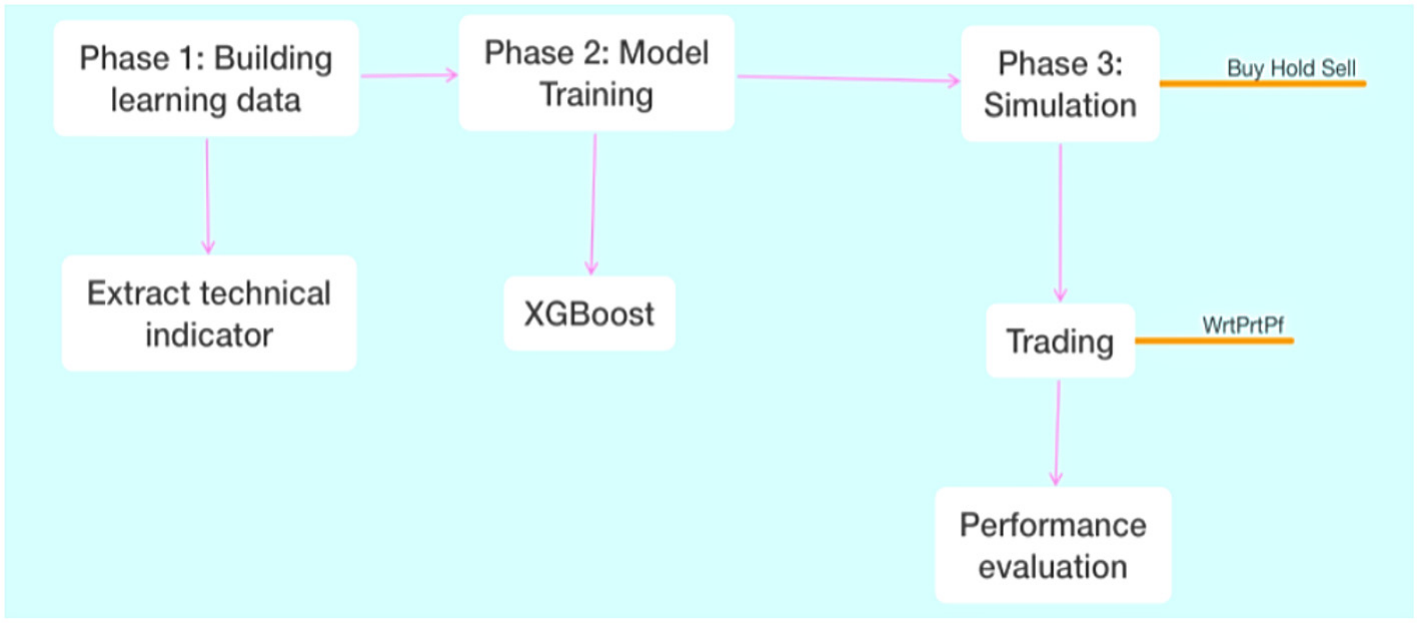
Flow chart for the stock market system (Li, 2023).

Significant features of stock price data, including non-linearity, non-stationarity, high noise, and substantial time variation, make accurate stock price prediction difficult. Technical analysis was the primary method employed in the early study, as it is in the current research. Among these, technical analysis combines typical market indicators, such as trading volume and stock transaction prices, to ascertain the trend of stock prices (Khalil et al., 2022). Researchers frequently use time series models to forecast stock prices. The generalized autoregressive conditional heteroskedastic (GARCH) model, the difference-integration moving-average autoregressive (ARIMA) model, and the vector autoregressive (VAR) model, a variant of the ARIMA model, are among the most frequently used models (Janiesch et al., 2021). Apart from the conventional econometric model, gray model, BP neural network, and fuzzy theory, numerous other approaches have been applied to stock price prediction. This study proposes a marking technique called N-cycle min-max (NPMM), which has been successfully applied in stock market analysis (Li M. et al., 2022). The NPMM tag model uses XGBoost, a non-linear machine learning model and C++ library, to enhance the performance of gradient boosting (a regression and classification technique) and to develop the transaction system, thereby achieving transaction automation (Nadeem et al., 2021). The three stages of the study are depicted in Figure 8: simulation, model training, and data accumulation. The author attempts to create learning data for the system in phase one. The writers examine the technical indicators to achieve this. The technical indicator is a statistical tool used to forecast stock market movements based on previous price data. Murphy (1999) suggests the following formula for the *N*-period Min–Max labeling: Lt^1+^ = 0 if Ct = *N*_max_, Lt^1+^ = 1 if Ct = *N*_min_. The labeling feature, which provides labels only for the minimum and maximum periods within the window, is demonstrated by the formula (Yoosefzadeh-Najafabadi et al., 2021). The window period is the time frame defined by the labeling approach, as the study employs the window-based labeling suggested by Sezer and Ozbayoglu (2018). Because the NPMM labeling evaluates the minimum and maximum window periods, it is insensitive to slight variations. The authors then proceed to the following stage of their study, which involves training the model using XGBoost. The authors create label predictions to automate the stock market as they go from phases 1 and 2 to phase 3 (Zhu et al., 2022). The authors utilize the XGBoost model they have trained to generate stock market signals and automate stock market processes in phase three of the simulation. The authors evaluate performance after automating stock market activities (Otchere et al., 2020). Data ingestion, feature engineering, model training and assessment, and model deployment are all included in a fully automated stock market machine learning pipeline. Its unique feature is that it constantly learns. As fresh market data comes in almost in real time, the system re-educates itself to make more accurate forecasts (Xiao and Ke, 2021). It is possible that many algorithms may be executed in parallel within such a system, and that the algorithm performing best would be selected based on the market's volatility (Prata et al., 2024).

The goal of the performance evaluation is to assess the trading system created using the authors' suggested NPMM labeling approach. A practical assessment is necessary to ensure that the purchasing and selling system designed with the NPMM labeling approach functions correctly. Using learning data figures, win ratio (Wr), payout ratio (Pr), and profit factor (Pf), as shown in Figure 3, the author assesses the trading system (Türkbayragi et al., 2022).

### 4.5 Management and optimization of portfolios

By leveraging risk assessment, real-time market insights, and predictive analytics, machine learning in portfolio management and optimization empowers investors to make data-driven decisions (Li and Pan, 2021). AI models, such as sentiment analysis, deep learning, and reinforcement learning, can be integrated into investment strategies to dynamically adjust to shifting market conditions. To anticipate future returns, minimize risk exposure, and identify trends in financial data, machine learning algorithms facilitate asset allocation optimization (Jabeur et al., 2021). Furthermore, sophisticated methods that assess millions of possible portfolio scenarios, such as Monte Carlo simulations and Bayesian optimization, improve decision-making (Gao et al., 2020). With the growing complexity of financial markets, ML-driven portfolio management gives institutional and retail investors a competitive edge by enhancing diversification, automating rebalancing, and offering real-time risk monitoring (Henrique et al., 2018). Integrating transformer-based models, such as GPT and BERT, with financial data analysis is a recent development in machine learning for portfolio management and optimization. By evaluating vast volumes of unstructured data, including financial news, investor sentiment, and earnings reports, these models enhance decision-making by providing more precise forecasts of market movements (Li et al., 2019).

Furthermore, dynamic portfolio rebalancing is now utilizing Deep Reinforcement Learning (DRL), in which AI agents continually interact with real-time market data to develop the most effective investment strategies (Bansal et al., 2022). The application of quantum computing for faster portfolio optimization is another innovation that enables investors to manage complex risk-return trade-offs (Long et al., 2020) more effectively. AutoML (Automated Machine Learning) is also being utilized by hedge funds and other financial organizations to develop flexible investment plans without the need for laborious manual model modification. By making portfolios more flexible, effective, and resilient to market fluctuations, these technologies are transforming the way they are managed (Singh and Srivastava, 2016).

Risk assessment is crucial to effective portfolio management, ensuring optimal asset allocation. The use of machine learning to evaluate portfolio risk and diversification has been the subject of several studies. The effectiveness of machine learning approaches for risk estimation was demonstrated by (Khojasteh and Daliri 2021), who proposed a portfolio risk prediction model based on extreme gradient boosting. A balanced and diversified portfolio is ensured by investors making well-informed selections thanks to practical risk assessment (Vijh et al., 2020). Examining the effects of various predictive horizons is essential for portfolio prediction. Both short-term and long-term predictive models have advantages and disadvantages. For instance, long-term models must account for shifting market dynamics over extended periods, whereas short-term models are susceptible to market fluctuations. Research on the effects of predictive horizons on portfolio performance found that combining short- and long-term predictions yields better results (Mehtab and Sen, 2020). Contemporary portfolio optimization incorporates risk-adjusted return maximization, dynamic rebalancing, scenario testing, and other techniques beyond the mean-variance framework of static theory. Its novel feature is that it incorporates reinforcement learning, meaning the portfolio can learn over time the best allocation strategies through experiments across thousands of market conditions. This enables the adjustment of assets beforehand to optimize profits and mitigate risk across a range of economic scenarios (Sahu et al., 2023; Fan and Zhang, 2024).

**Figure 9:**
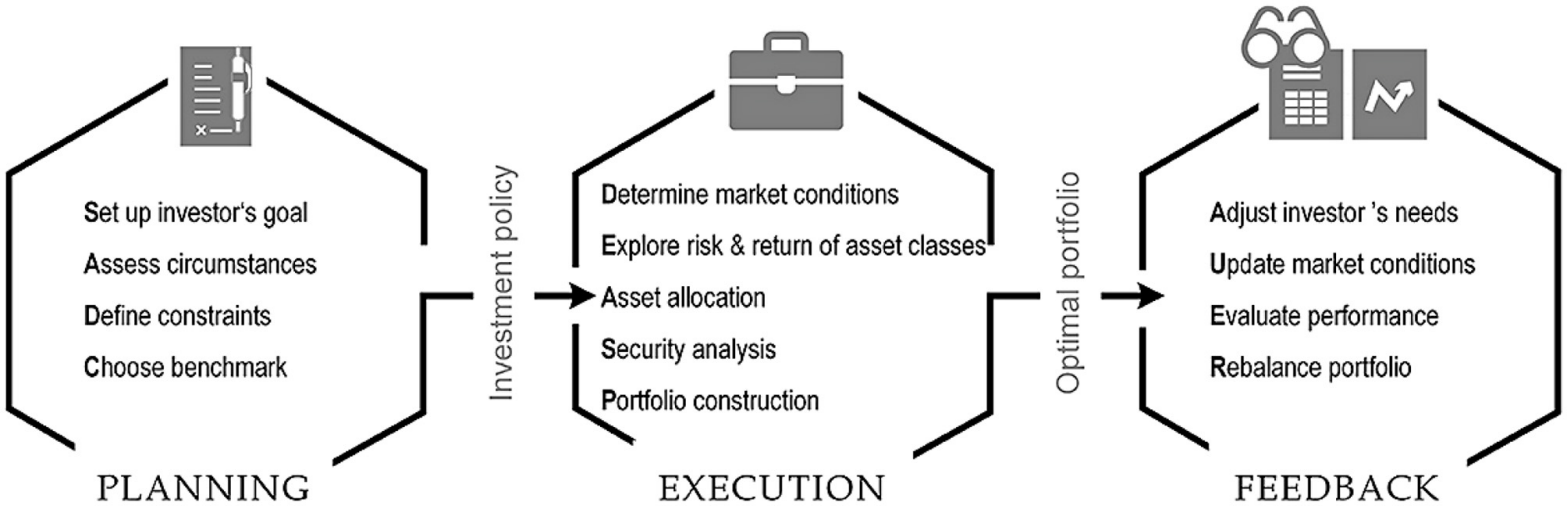
Procedure for managing a portfolio (Sutiene et al., 2024).

Portfolio management is the ongoing process of building portfolios based on the risk and reward levels investors choose, and then modifying them over time to optimize returns. Figure 9 illustrates the three succeeding stages of this process: planning, implementation, and feedback. The method starts with the planning layer (Strader et al., 2020). An institutional customer, such as a pension fund or a wealth management client, is the asset owner and directs an asset manager to manage a specific portfolio in accordance with an investment policy. This mandate is the investment policy. The requirements, conditions, and limitations the client must meet to achieve a particular reward target at a given risk level are outlined (Ince and Trafalis, 2008). This policy includes Strategic Asset Allocation (SAA). Typically, the top and lower bounds of the asset class allocation define the SAA. It is also necessary to identify risk capacity and risk tolerance. The execution layer is the second tier of the portfolio management procedure. Determining the general macroeconomic circumstances across nations and asset classes and investigating the risk-and-return characteristics of these asset classes are the first steps in the execution process (Chen J. et al., 2023).

The capital distribution across countries and asset classes, also known as tactical asset allocation, is determined by this approach. To build the total portfolio and execute the required trades, security analysis enables the selection of individual securities within each asset class cross-sectionally. The feedback layer then assesses historical performance, updates market conditions, determines whether the investment policy is still applicable or needs to be modified, and rebalances the portfolio after it has experienced the market dynamics of an investment period (Hiransha et al., 2018). The banking industry is among the many areas that artificial intelligence (AI) has upended in recent years. AI approaches can enhance portfolio management in various ways, addressing the limitations of traditional portfolio design methods and expanding the potential for alpha generation. For example, machine learning (ML) can be used to develop algorithms that forecast asset prices by learning from past experiences. One of the most promising methods for developing a dynamic, sequential portfolio optimization theory is reinforcement learning (RL) (Chen C. et al., 2023). With new market news, text mining and sentiment analysis can improve portfolio management. The development of a well-diversified portfolio is enhanced by dimensionality reduction techniques that identify hidden components across a wide range of asset prices. With a limited collection of assets, deep learning may create a portfolio that replicates an index or directly optimizes an investment portfolio (Li Y. et al., 2022).

## 5 Advancement in AI for stock price forecasting

**Figure 10:**
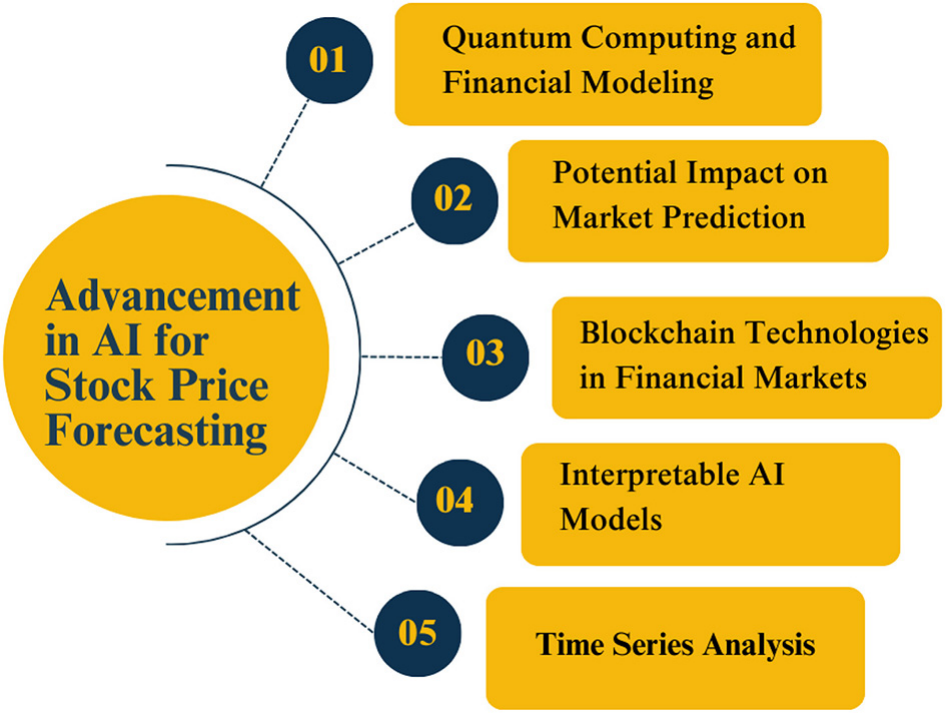
Some advances in AI for stock price forecasting.

In recent years, the application of AI in stock price prediction has demonstrated considerable promise. Compared to traditional regression and analytical models, deep learning models, such as neural networks, have greater potential to leverage vast volumes of data to identify essential and subtle patterns (Oyewole et al., 2024). Five advancements in AI for stock price forecasting are shown in Figure 10 as follows: (i) Quantum Computing and Financial Modeling; (ii) The Potential of AI in Market Prediction; (iii) The role of Blockchain in Financial Markets; (iv) Interpretable AI Models; and (v) Time Series Analysis.

Here are a few significant advancements:

### 5.1 Quantum computing and financial modeling

In domains such as financial modeling, quantum computing is emerging as a disruptive force, with potential applications in portfolio optimization, a challenging problem in investment management that seeks to maximize returns while minimizing risk, particularly as the number of assets increases (How and Cheah, 2023). The exponential growth of feasible asset combinations makes classical approaches, such as Markowitz's mean-variance optimization, computationally demanding. It may be possible for quantum algorithms, particularly the Quantum Approximate Optimization Algorithm (QAOA), to overcome this difficulty by processing numerous solutions simultaneously via superposition states of qubits, thereby reducing the time required to identify the ideal portfolio (Jha et al., 2025b; Ajlouni, 2024). Large-scale portfolio optimization problems greatly benefit from the use of QAOA's quantum circuits to search the solution space and iteratively update the quantum state to minimize a cost function representing the portfolio's risk-adjusted return (Carrascal et al., 2023).

The ability to handle vast amounts of data at rapid speeds is at the heart of quantum computing. Qubits, the building blocks of quantum computers, can exist in multiple states simultaneously rather than just one. Due to these capabilities, quantum computers may be able to run financial models and simulations in a timeframe likely unmatched by traditional computers. This means that, compared to humans, it can digest vast amounts of historical data, market patterns, and financial data quickly enough to deliver better, faster outcomes for stock price predictions (Bravyi et al., 2022; Yuan et al., 2020). In the context of artificial intelligence, feature selection is crucial because it determines the input variables for the stock forecasting model.

Additionally, the size of feature space is expanded by quantum computing, which is capable of handling more variables than current methods, including sentiment analysis of various social media platforms, macroeconomic aspects, and fundamental company issues. This feature enables the specification of additional variables that can be included in the models, leading to more accurate predictions (Vashishth et al., 2025; Mousa and Shirazi, 2024; Doosti et al., 2024). Quantum machine learning (QML) is a concept that combines conventional machine learning techniques with quantum computing to improve stock price predictions. Moreover, quantum-inspired classifiers may enhance the training of neural networks and other models, thereby improving prediction accuracy. For instance, quantum-realized support vector machines (SVMs) can handle larger datasets and yield results that are much more in line with market realities (Priyadarshini, 2024; Yung et al., 2020).

New developments in data security, driven by quantum computing, are also significant for the financial industry. Thus, the enhanced features of quantum cryptography protect critical data, including trading techniques and financial information, from cyber criminals. Given that the data is sensitive and can be kept private in the database, this security enables financial institutions to develop complex forecasting models for goods, services, and scholarships without exposing them to unauthorized individuals (Baseri et al., 2024; Divyashree, 2024). High speed is a key feature of quantum technologies, essential for generating accurate forecasts of the erratic stock market promptly. Applications built with quantum technology can quickly understand shifting markets and make adjustments based on the latest data, unlike older systems. This agility allows the opponent to generate noticeably higher average returns and is especially useful in high-frequency trading (Alaminos et al., 2022). To properly comprehend the model used to present financial aid outcomes, the risk must be assessed. Because quantum computers can simulate conceivable market conditions and probable outcomes considerably more quickly than conventional computers, they can also be used for high-risk assessments. Financial organizations can achieve far more effective risk management by leveraging quantum algorithms, which offer a higher likelihood of being prepared for periods of volatility (Nafiu et al., 2025). Thus, the ability to identify patterns and anomalies in massive datasets is another notable application of quantum computing. It is also crucial to remember that there are often many weak trends in financial markets that traditional indexes struggle to identify. This study found that quantum systems may facilitate correlations across multiple equities, enabling traders to make informed judgments about stock price movements (Park et al., 2021). In financial modeling, the use of quantum computing is innovative because its outcomes outperform the exponential speed of a traditional computer in solving problems and simulating. Quantum Monte Carlo and Quantum Approximate Optimization Algorithms (QAOA) are two quantum algorithms that can be used to address high-dimensional problems in risk assessment, option pricing, and portfolio optimization that cannot be computed using traditional methods. As never before, it enables financial models to handle massive amounts of data and quickly and accurately disclose market patterns (Muhammad et al., 2024; Rane N. et al., 2024; Fan et al., 2024).

Recent experimental studies on current quantum hardware, such as those using the Quantum Approximate Optimization Algorithm (QAOA) for portfolio optimization, highlight the nascent stage of this technology. While demonstrating proof-of-concept, these studies report that solutions from noisy intermediate-scale quantum (NISQ) devices often do not yet surpass the performance of highly optimized classical algorithms like Gurobi on practical problem scales, primarily due to qubit coherence limits and error rates (Zheng J. et al., 2024; Carrascal et al., 2023; Nguma et al., 2024). The potential is undeniable, but claims of quantum supremacy for financial modeling are premature; the immediate impact is more likely in quantum-inspired classical algorithms and specialized risk simulations (Mishra et al., 2024; Doosti et al., 2024).

**Figure 11:**
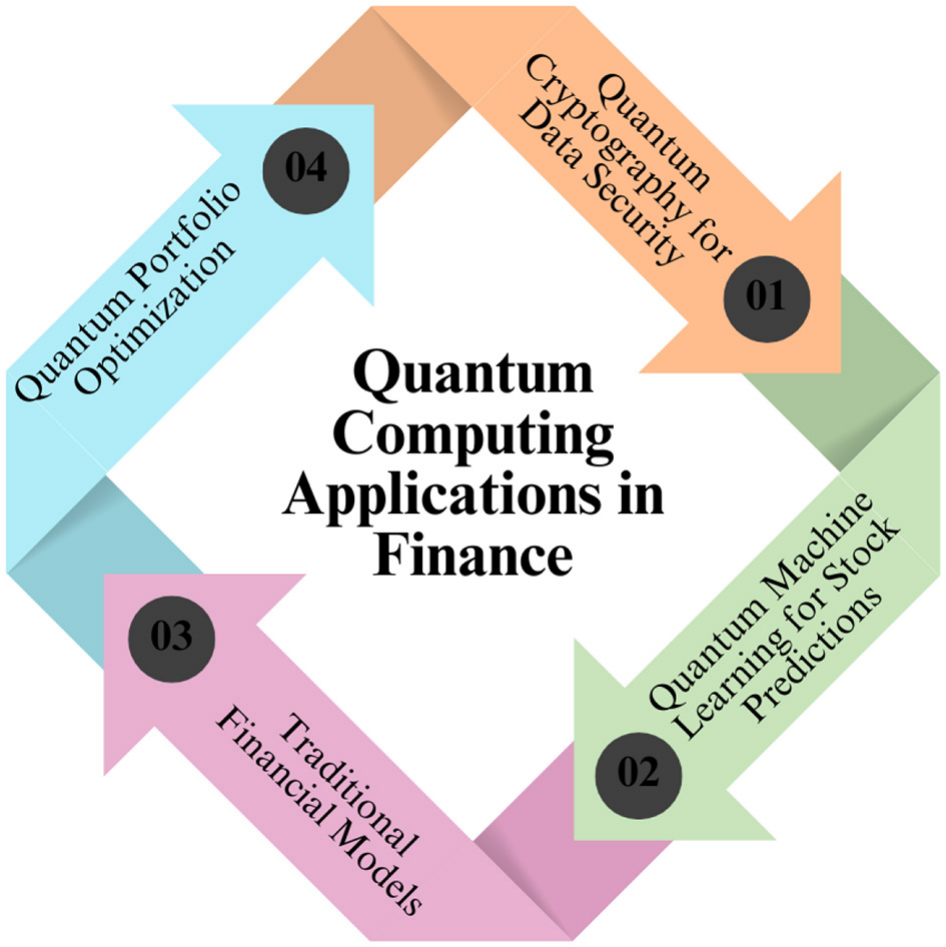
Quantum computing applications in finance.

Quantum Cryptography for Data Security, Quantum Machine Learning for Stock Predictions, Quantum Portfolio Optimization, and Traditional Financial Model are the four portions of the circulation model used in Figure 11 to illustrate how quantum computing pertains to finance.

### 5.2 Potential impact on market prediction

Enhancements in processing speed, predictive accuracy, and the ability to manage vast datasets with unprecedented efficiency are just a few ways quantum computing may impact market prediction. By enabling quicker and more thorough evaluations of market possibilities, quantum algorithms, particularly in portfolio optimization, have the potential to transform financial forecasts and enhance the flexibility of investment strategies (Singh et al., 2024). By improving parameter tuning and addressing issues such as overfitting in conventional machine learning models, the exponential computational power of quantum computing improves prediction accuracy. It is ideal for analyzing large datasets to identify subtle trends and refine parameter settings, thanks to its ability to process enormous volumes of data simultaneously (Manushree et al., 2024). It is also ideal for analyzing large datasets to identify subtle trends due to its ability to process massive volumes of data simultaneously (Sajjan et al., 2022). Furthermore, the promise is demonstrated by quantum machine learning, which leverages quantum physics to accelerate model training and improve stock price forecasts by enabling faster, more efficient convergence. However, incorporating quantum computing into market prediction is challenging due to existing limitations in qubit count, coherence durations, and error rates, as well as the need for specialized knowledge (Nguma et al., 2024).

Modern technology has enabled AI algorithms to provide insights into real-time market data in a remarkably short period. By reacting to the market quickly, closing trades promptly, and seizing fleeting opportunities, this characteristic gives traders flexibility in how they respond. Because transactions are executed in milliseconds based on algorithms rather than an investor's intuition, using algorithms to trade regularly can affect the market (Jabbar et al., 2019; Cohen, 2022). The development of artificial intelligence has had a significant impact on algorithmic trading, enabling the combination of multiple specified methods into more complex algorithms that aid in market situation adjustment. Machine learning (ML) models are beneficial for trading, as they can adjust algorithmic parameters based on past data and optimize entry and exit points for each transaction. Better trading tactics that improve the capacity to learn and adjust to its surroundings are a result (Dakalbab et al., 2024; Mashrur et al., 2020). AI-powered stock price forecasting tools help firms manage risk more effectively. It is a method by which machine learning algorithms can identify trends associated with hazards, such as bears and volatility. By predicting when the market is likely to decline, these models help investors minimize losses by discouraging them from purchasing riskier securities during specific periods (Singh et al., 2022).

Additionally, AI enables the creation of customized investment options tailored to individuals' risk tolerance and personal investing preferences. Therefore, AI systems can assist individual investors and institutions in developing and implementing comprehensive, customized investment plans tailored to their unique risk management needs and preferences, leveraging massive datasets (Singh et al., 2025). Technological advancements have increased demand for RNS and facilitated access to high-quality financial research for a diverse range of investors, including private individuals. The information and projections that were previously only accessible to institutional investors can now be obtained through new artificial intelligence-based platforms, such as IEnumerator. This may result in more individuals actively trading and investing in the financial market, thereby increasing its diversity (Du and Xie, 2020; Munoko et al., 2020). Although using AI to predict stock prices may enhance human connections, it also poses risks and ethical concerns. The emergence of highly sophisticated trading systems may lead to market manipulation if algorithms are utilized maliciously.

Additionally, the process of AI decision-making lacks transparency, making it difficult for market players to comprehend how and why specific forecasts are generated (Felzmann et al., 2020). The description of how new computing paradigms, particularly the use of quantum and advanced AI systems, may result in significantly improved market forecasts in terms of timeliness and accuracy is hence the unique selling point. The new approaches focus on updating data almost instantly and on combining macro- and micro-based trade data with alternative data. This allows the prediction models to react instantly to changes in liquidity, geopolitical events, or economic shocks (Zheng J. et al., 2024).

**Figure 12:**
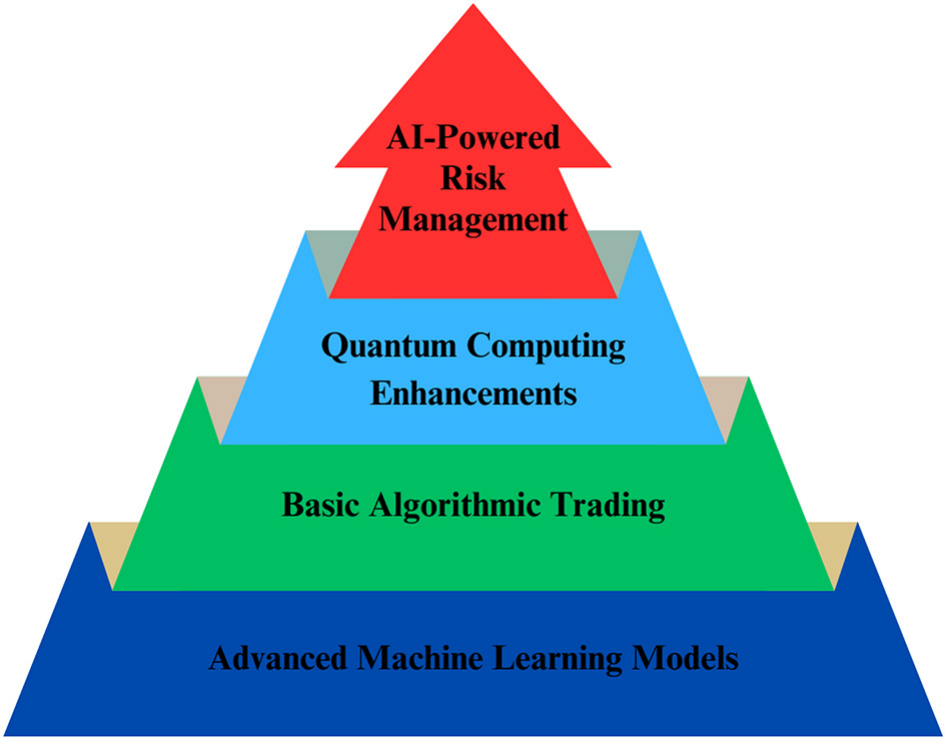
Technological advancements in market prediction.

Figure 12, which precisely explains the stages of technological advancement in stock exchange prediction, is titled “Technological Advancements in Market Prediction.” Advanced machine learning models are the primary key component of big-data systems, serving as the foundation for nearly all applications of predictive analytics today. The principles of trading with algorithmic systems are covered in Basic Algorithmic Trading. Advances in quantum computing aid in data processing and enhance existing financial modeling techniques. This involves mitigating corporate risks by applying artificial intelligence to decision-making.

### 5.3 Blockchain technologies in financial markets

Decentralized trading platforms, also known as decentralized finance (DeFi) platforms, are a revolutionary development in the financial markets. They utilize smart contracts and blockchain technology to eliminate intermediaries, making trade more transparent and efficient (Ozili, 2022). Distributed ledgers underpin these systems, ensuring the safe and immutable storage of transaction records, while smart contracts streamline trade execution, reducing costs and increasing speed (Liu et al., 2024). AI integration, primarily through AI-driven trading bots, further enhances DeFi systems by evaluating vast volumes of market data in real time, identifying opportunities, and executing trades autonomously (Amirzadeh et al., 2022). Open-source AI models, such as DeFi, encourage transparency in contrast to proprietary algorithms in centralized settings. AI also increases liquidity by democratizing market access worldwide, enabling large transactions, and aggregating liquidity from multiple sources, making trading operations accessible to everyone with an internet connection (Alamsyah et al., 2024).

These systems are equipped with smart contracts that enable automated transactions in response to predefined criteria. Instead, when specific market signals identified by AI models are met, contracts may execute transactions without human intervention. To prevent losses, a smart contract can be set up to sell the asset itself if an AI system predicts a decline in the asset's price. AI and smart contracts working together enhance trading speed and reduce costs, enabling traders to respond quickly and adapt to market changes (Kirli et al., 2022; Taherdoost, 2023). The process of transforming equities into tokens that are stored on a network is known as tokenization. This may simplify the process of purchasing or selling tokenized assets and create opportunities for speculation, thereby increasing efficiency in financial markets. From an artificial intelligence forecasting perspective, tokenized assets may provide real-time information on trade volume and prices. Artificial intelligence (AI) models may enhance market analysis, leading to more accurate emission estimates and informed investment planning that considers other asset types (Ciriello, 2021; Makarov and Schoar, 2022). Features of this, such as its tamper-proof ledger and decentralization, encourage confidence in the market's product flow. According to reports, every transaction is recorded in a public registry that anyone connected to the network can view and review at any time, if necessary. By including this openness, the likelihood of stock market fraud and manipulation is reduced. To enhance trade decision-making, AI models that use transparent data will enable stakeholders to gain more trustworthy insights than those that rely on opaque data sources that may not be verified or vetted (Rane et al., 2023a; Pillai, 2023; Sethi and Mahadik, 2025). Due to the exemplary algorithms of artificial intelligence that can process vast amounts of data stored in, from trades and orders to sentiment reflections in social media platforms, it is possible to identify several otherwise latent patterns and relationships that are not possible with more conventional approaches. The combination of blockchain and AI opens the door to more advanced market analytics that can alter the status quo. This is because conclusions drawn from AI-assisted data analysis provide traders with a deeper understanding of the market and, consequently, better stock price predictions (Fior et al., 2022; Hajj and Hammoud, 2023). This technology offers novel approaches to record transactions in a transparent, secure, and immutable manner, reducing counterparty risk and enabling faster settlements. It can support decentralized clearing systems, tokenized asset markets, and real-time audits in addition to cryptocurrency trading (Mukherjee et al., 2021). By combining this technology's distributed ledger with predictive analytics, their solution enables the prediction of market trends based on wallet activity, transaction flows, and smart contract interactions (Rane N. L. et al., 2024).

**Figure 13:**
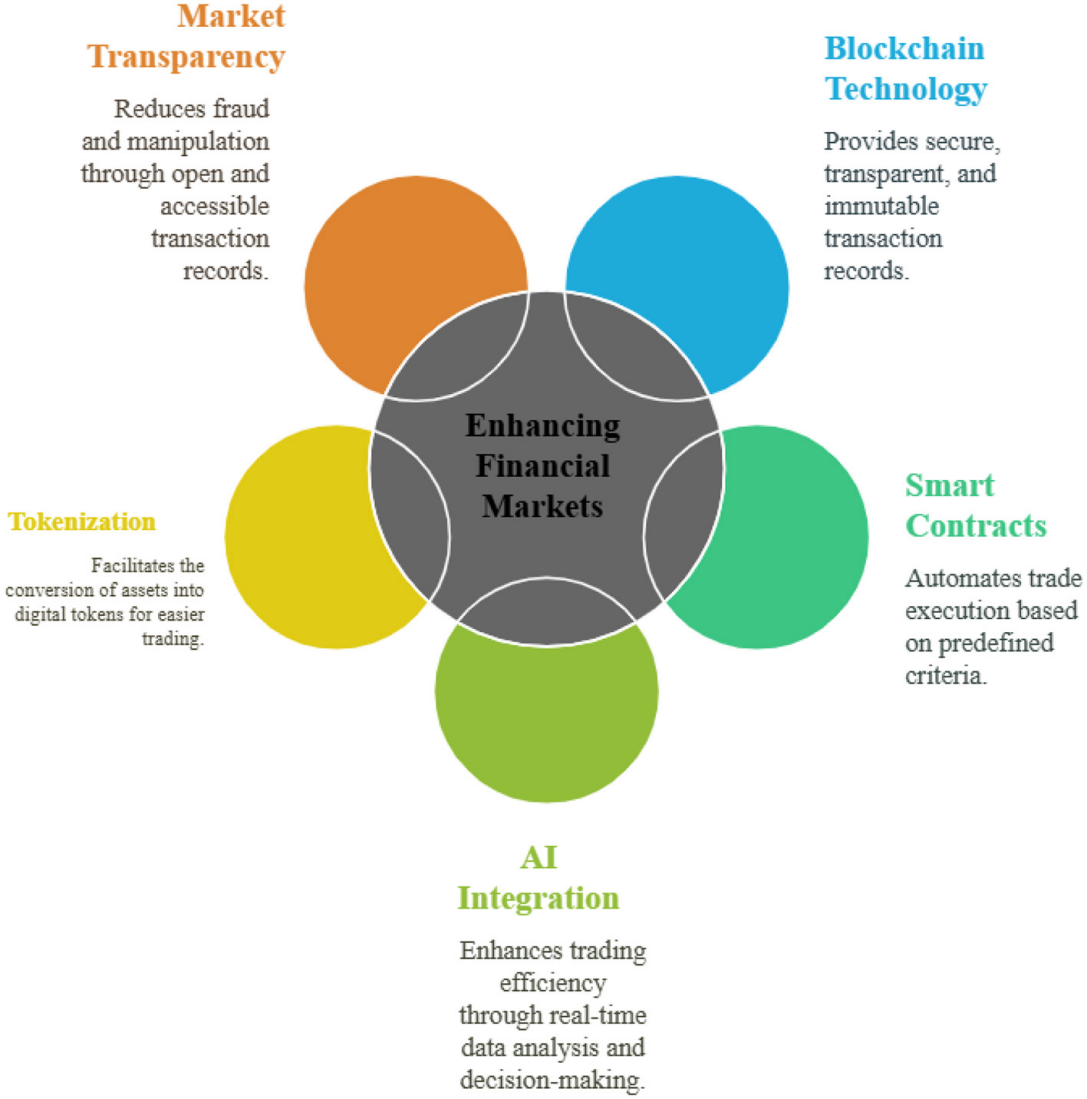
Enhancing financial markets.

Figure 13 illustrates the various technologies that work in tandem to enhance the financial markets. Around the center are nodes such as Market Transparency, Blockchain Technology, Smart Contracts, AI Integration, and Tokenization. At the center is the current strategic development plan, called “Enhancing Financial Markets.” Each node illustrates a different way in which these technologies improve market efficiency, security, and transparency.

### 5.4 Interpretable AI models

The stock price has been predicted using machine learning, a branch of artificial intelligence, and recently, interpretable AI models have gained attention. In addition to forecasting stock prices, these models also predict the underlying reasoning behind them, which is particularly significant in the financial industry, where transparency is desired (Minh et al., 2021). Since financial trading is often associated with high-risk investments, making models more straightforward to understand enables investors to assess their predictive reliability more effectively. With this information, analysts can determine the elements that influence choices and identify the associated risks. Financial professionals must understand the physics of using AI for predictive modeling to boost consumer confidence (Maqsood et al., 2019). Interpretable AI often uses feature importance analysis to assess the impact of different parameters on the model's predictions. To enable investors to focus on the key market elements, SHAP (Shapley Additive Explanations) and LIME (Local Interpretable Model-agnostic Explanations) can provide insights into the most significant characteristics that influence the forecasted stock price (Rane et al., 2023b). Despite being very straightforward methods, linear models are simple to understand. They offer estimates that clearly show a relationship between the stock prices and the predictor factors. To facilitate interpretation, regression analysis helps practitioners estimate the relative importance of each prospective element. However, there are certain drawbacks to building linear models, chief among them that they cannot account for non-linear interactions (Del Vitto et al., 2023; Singh and Khushi, 2021).

Standard decision trees are straightforward to understand because the function repeatedly separates the data based on feature values, yielding rules that are always clear and concise. Investors can see how input characteristics contribute to predicted values, as these models are also readily visualized. As a result, pruning and other such changes may enhance performance without sacrificing the degree of interpretability that dynamic stocks may demand (Sarker, 2021). To increase accuracy, specific algorithms, such as Random Forests and Gradient Boosting, build an ensemble of decision trees. Interpretability can be achieved to some degree through feature significance metrics, though they are not as easy to comprehend as single decision trees. These techniques help predict stock prices and meet both accuracy requirements and the need for additional model knowledge (Kumbure et al., 2022). Several functional models of Explainable Boosting Machines have been developed as novel boosting algorithm variants that prioritize interpretability without sacrificing the performance of the resulting models. It combines aspects of boosting techniques with generic additive models to produce understandable results. This enables investors to observe how certain characteristics interact with one another and how specific changes to the independent variable impact the outcomes (Block et al., 2020). Direct incorporation of interpretations into prediction models has been the subject of recent studies. For example, attention layers that emphasize pertinent input sequences are an interpretability characteristic included in several deep learning systems. This emerging field is fascinating because it combines the openness required for financial applications with high-performance models (Lisboa et al., 2023). Since one is aware of every effect the model will have in a given market, interpretable models may be more resistant to such changes than other models. By prioritizing features and monitoring the model's effectiveness, investors can adjust their approach when new information becomes available, thereby reducing risk (Awijen et al., 2024). The possibility of achieving both high prediction accuracy and decision-making transparency is a key feature of interpretable AI models in the financial industry. Explainable approaches, such as SHAP values, LIME, or attention mechanisms in deep neural networks, may be used to ensure that traders, risk managers, or regulators can understand the logic behind a given forecast. Such compromises between performance and intelligibility improve adoption, faith, and privacy in high-stakes financial situations (Wang et al., 2024b).

Explainable AI (XAI) has become an essential component of financial forecasting systems because regulatory bodies increasingly require transparency in automated decision-making. Although deep learning and reinforcement learning models demonstrate strong predictive performance, their opaque internal structures limit institutional trust and complicate compliance with audit, interpretability, and accountability requirements (Tuarob et al., 2021). Techniques such as SHAP, LIME, Grad-CAM, and attention-weight visualization offer partial transparency by identifying influential features, yet they struggle to fully capture the temporal complexity and interdependencies present in financial time series (Smith and O'Hare, 2022). As a result, the lack of precise interpretability mechanisms remains a major barrier to the adoption of deep learning-based forecasting models in high-stakes financial environments (Zhu et al., 2021).

**Figure 14:**
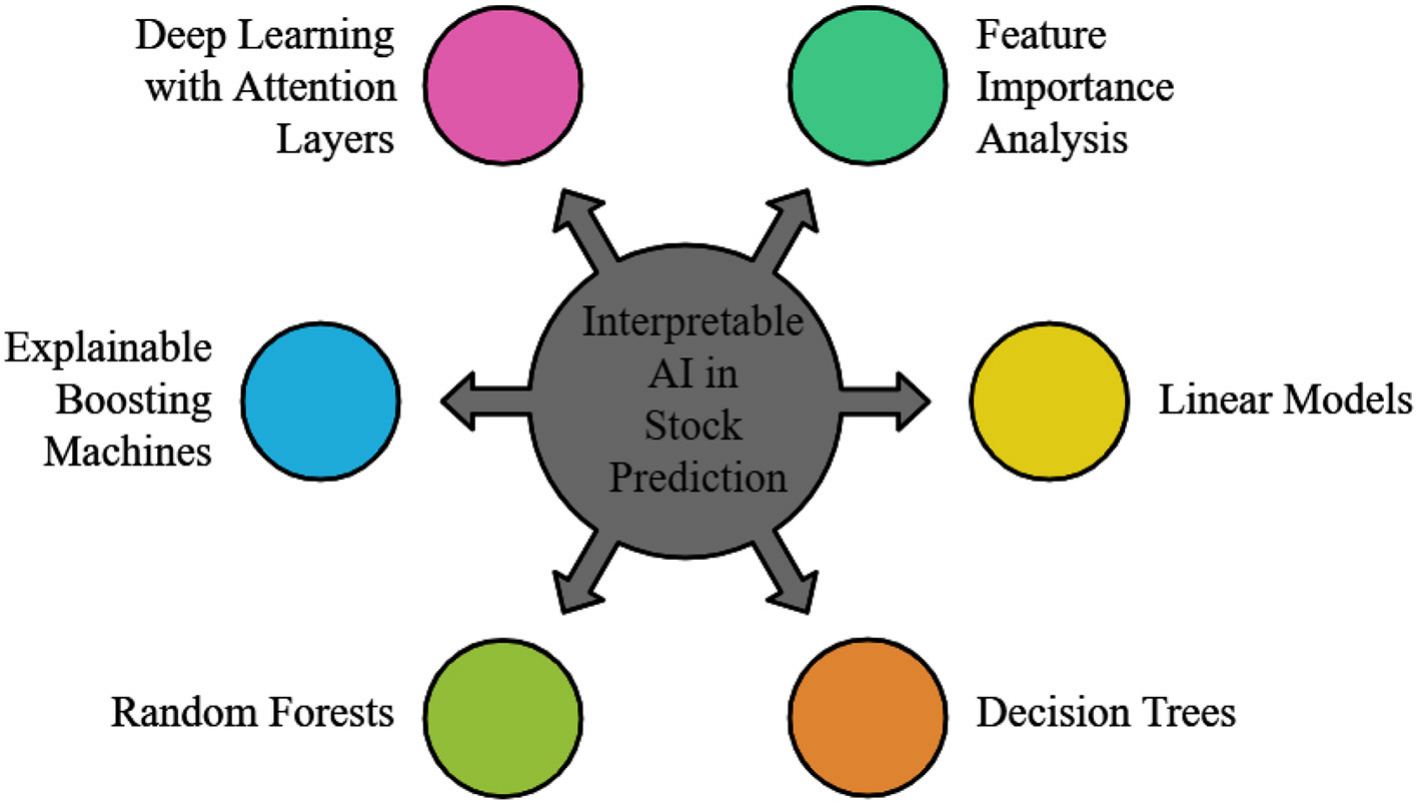
Interpretable AI in stock prediction.

Some machine learning and artificial intelligence techniques used to forecast stock prices are illustrated in Figure 14, which falls under the broad category of interpretable AI. To increase the explainability of decision-making in models used to predict stock market movements, it is crucial to discuss Deep Learning with Attention Layers, Feature Importance Analysis, linear models, Decision Trees, Random Forests, and Explainable Boosting Machines.

### 5.5 Time series analysis

Time series analysis is a method for predicting the price of a specific stock by examining data gathered and organized in chronological order. Stock prices have been successfully incorporated into international final analysis techniques for stock price forecasting, primarily due to the availability of large datasets. Artificial intelligence advancements have ushered in a new era, making it easier to examine time series data and providing more accurate forecasts that enhance investment strategies (Tuarob et al., 2021; Smith and O'Hare, 2022). Historically, time series analysis has been conducted using methods such as ARIMA (Autoregressive Integrated Moving Average) and exponential smoothing, which utilize data patterns to predict future events and identify patterns in various fields of study. These methods have been helpful in other areas, but they are unable to adequately solve complex, non-analytical problems, such as those found in financial markets. However, with the help of artificial intelligence, this has been accomplished (Zhu et al., 2021). Machine learning (ML) in time-series analysis has been shown to improve stock price forecasting significantly. Such algorithms may uncover specific patterns and analyze vast amounts of historical data more effectively than human mathematical models. The employment of approaches such as ensemble methods, decision trees, and vector support has been suggested for linguistic variables and their interactions (Sonkavde et al., 2023). When it comes to using AI to predict stock prices, RL is one of the most widely used methods, often outperforming conventional methods. With an emphasis on selecting the best option among alternatives, trading tactics in RL are learned from the environment. These models may be used to supplement time-series analysis by simulating trading conditions and determining the best methods to maximize profits while balancing multiple risks (Gao et al., 2022; Sevastjanov et al., 2023).

Advances in feature engineering have improved the input used to create time series models. Because analysts now employ a variety of features, including firm-specific elements, macroeconomic data, and non-traditional data sources such as aerial photos and transaction records, complexity has increased in the modern period. For AI models to provide more accurate predictions, it is crucial to include such enriched datasets (Akroyd et al., 2023). However, given the present situation, appropriate backtesting and validation are essential when using AI to estimate stock values. Generally, backtesting involves evaluating models using historical data. Numerous computations and analyses can be performed to determine how models perform under specific market conditions. These methods help improve the models, enabling investors to view them more accurately (Kumar et al., 2020; Kraus et al., 2019). Time series analysis techniques for stock price forecasting have advanced significantly with the availability of extensive data. Real-time information, additional datasets, and high-frequency trade data all help AI models analyze large amounts of data quickly. This feature has several advantages, including improved forecasting and the ability to react to market fluctuations, which enhances trading strategies (Kurani et al., 2021). Thus, it is evident that the use of AI for stock price prediction raises current ethical concerns in its implementation. Accountability in FinTech is further underscored by skepticism regarding the operation of these technologies, particularly in areas such as data security, algorithms, and stock market manipulation.

To ensure that such technology enhances the market, models must utilize it ethically (Roszkowska, 2020). The future of AI in time-series analysis for stock prices appears promising, given that past prices are a prevalent component of stock price forecasting. New technologies, particularly quantum computing, offer promising prospects for more extensive and advanced architectures and significantly faster data analytics. Furthermore, the use of AI in conjunction with blockchain technology may enhance the security and quality of the data presented, thereby improving the accuracy of the forecast (Farahani and Hajiagha, 2021; Wu et al., 2021b). Recent developments in time series analysis employ deep learning architectures such as Transformer networks and Temporal Convolutional Networks (TCNs) rather than traditional ARIMA or GARCH models. These new methods can capture seasonality in stock prices and other financial indicators, as well as long-term and non-linear connections (Bahoo et al., 2024). Its originality lies in the way hybrid time-series frameworks are designed to combine the flexibility of deep learning with the rigor of statistical models to produce more accurate forecasts amid noisy, turbulent market behavior (Kanaparthi, 2024b).

**Figure 15:**
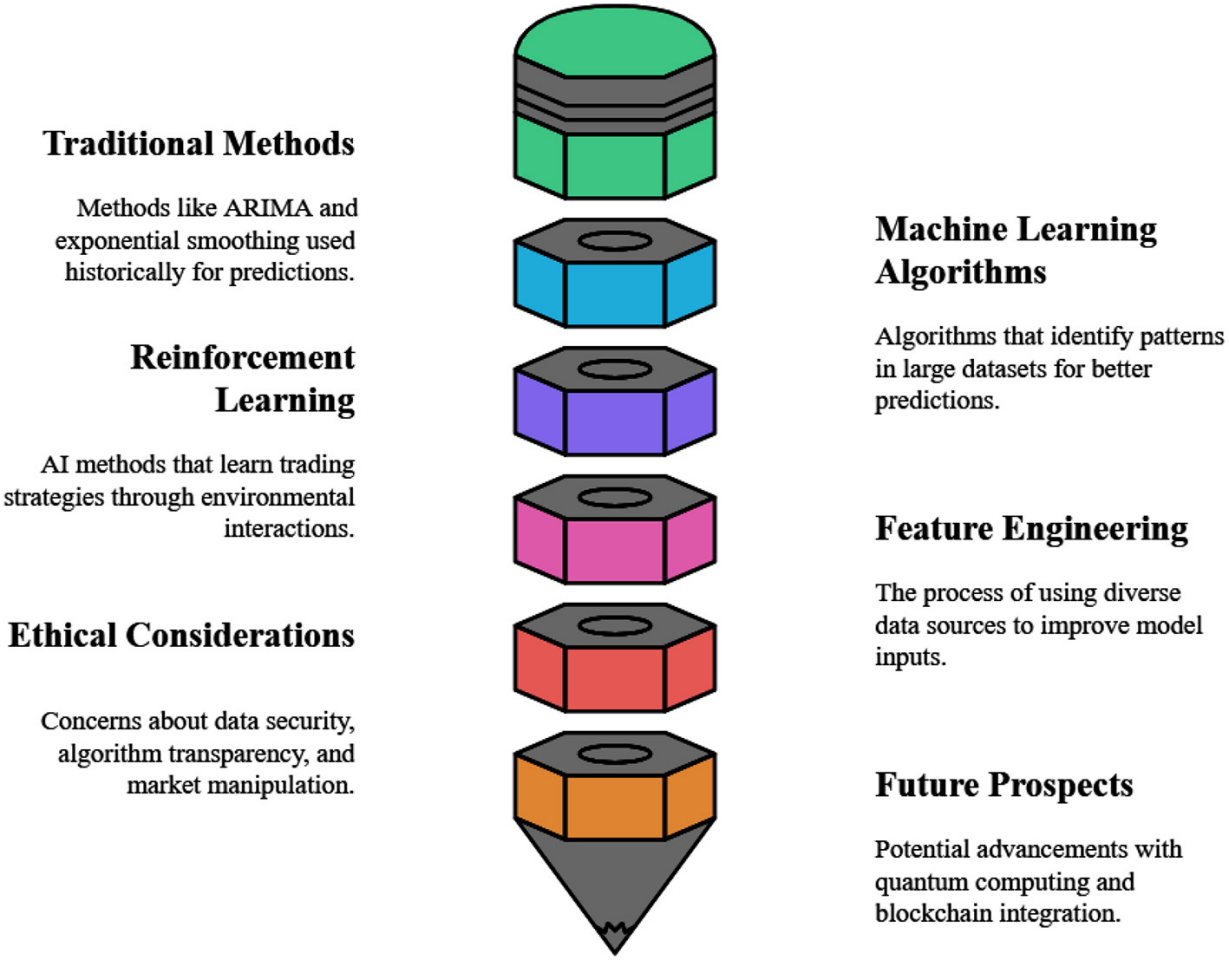
AI-enhanced time series analysis for stock prediction.

Additional examples of AI and machine learning applications in stock prediction are illustrated in Figure 15, with a focus on “interpretable AI.” It focuses on the concept of explainability in financial modeling and demonstrates how machine learning techniques, including Feature Importance Analysis, Deep Learning with Attention Layers, Linear Models, Decision Trees, Random Forests, and XGBoost, can recognize and contribute to the explanation of decision-making in AI models used for stock price analysis.

## 6 Challenges

The financial sector promotes the adoption of AI and ML, particularly for stock market predictions. However, a few challenges have made predicting stock values difficult (Khan et al., 2020). Since AI models are trained to learn and make predictions from the data provided, the quality of that data is crucial to their success. Numerous disparities can be observed in the historical stock price data, which may be influenced by a wide range of factors (Munappy et al., 2022). In addition, high-frequency trading data may include enormous volumes of trade-related information, and the cleaning procedure may result in format incompatibilities that require appropriate pre-processing for machine learning (Raddant and Kenett, 2020). Variability and sensitivity to changes in the overall economy, the political landscape, or the specific stock exchange are among the most universal characteristics of financial markets. Such circumstances introduce an element of unpredictability, making it challenging for machine learning algorithms to produce accurate future stock price predictions, as what has been learned may not hold in other circumstances (Pagnottoni et al., 2022). However, the main drawback of using machine learning models is that they often lead to overfitting, in which the model becomes overly reliant on the characteristics of the training set. Because the model fails to capture future data as intended, overfitting is detrimental in financial markets and often results in adverse outcomes. One of the most challenging decisions is determining the degree of model complexity, intending to achieve high generalization (Bhowmik and Wang, 2020).

Several operational, regulatory, and technological barriers hinder the real-world adoption of AI-driven forecasting systems in financial institutions. Sudden market regime shifts can trigger model drift, causing performance degradation that requires continuous monitoring and retraining (Martin and Nagel, 2021). Furthermore, inconsistent data quality, cybersecurity vulnerabilities, and exposure to adversarial manipulation increase the operational risks associated with deploying such systems in live trading environments. Regulatory frameworks such as the SEC guidelines and the European Union's AI Act impose strict requirements for model transparency, auditability, and risk documentation, which many deep learning and reinforcement learning models struggle to satisfy due to their black-box structure. Additionally, integration with legacy IT infrastructures found in banks and brokerage firms poses further constraints, collectively slowing the widespread deployment of advanced AI forecasting tools.

This is because the characteristics of models, such as biases in the chosen input variables, can affect the likelihood of accurate prediction. A more complex method should be employed to select the relevant variables, given the intricate network of relationships within the financial index system (Farzaneh et al., 2021). One must pay close attention to feature engineering and selection, as missteps in the selection process can lead to significant error rates, particularly in dynamic settings and financial applications (Theng and Bhoyar, 2023). International finance is a dynamic sector that constantly evolves, driven by technological advancements, regulatory changes, and shifts in global investor behavior. It also indicates that a model that performs well may not function well in circumstances other than those in which the data was gathered. This requires frequent model updates, which consume time and resources (Ahlstrom et al., 2020; Toma et al., 2020). The majority of machine learning models, and deep learning in particular, are opaque, making them difficult to understand. Because they are unable to understand how a political organization operates, this deficiency prevents financial stakeholders from making judgments. Increasing the model's interpretability without sacrificing its functionality is one of the difficulties that people or organizations encounter (Kumar et al., 2020). It is challenging to use AI models in the financial sector because, like other industries, it is subject to jurisdiction-specific regulations. Sometimes, especially after such tragedies attributed to algorithmic trading failures, authorities may restrict the use of data, trading techniques, or specific algorithms (Sahu et al., 2023). Along with regulatory issues, financial organizations must find ways to prevent market manipulation arising from algorithmic use. It is not easy to apply machine learning in today's environment, as it is often unclear which regulations or compliance standards must be met to introduce or develop this technology (Cao, 2022).

Despite advancements in machine learning over stock price forecasting, recent developments in artificial intelligence in financial market prediction are linked to the challenge of dealing with the highly volatile and non-linear nature of markets, as any unexpected event, geopolitical concerns, or abrupt macroeconomic changes can quickly cause the trend to deviate from long-term precedents and historical trends (Sarisa et al., 2024). The use of real-time data in an unstructured manner, such as news feeds and social media sentiment, without introducing noise, overfitting, sensitivity to back data, or a lack of interpretability, is a common problem with AI systems. Moreover, although additional ethical and legal concerns, such as algorithmic explainability, data security, and market manipulation risk, may make the live deployment of AI systems even more difficult, it may be challenging to guarantee even a sufficient, objective, high-quality dataset (Huang et al., 2023a; Srivastava et al., 2023).

**Figure 16:**
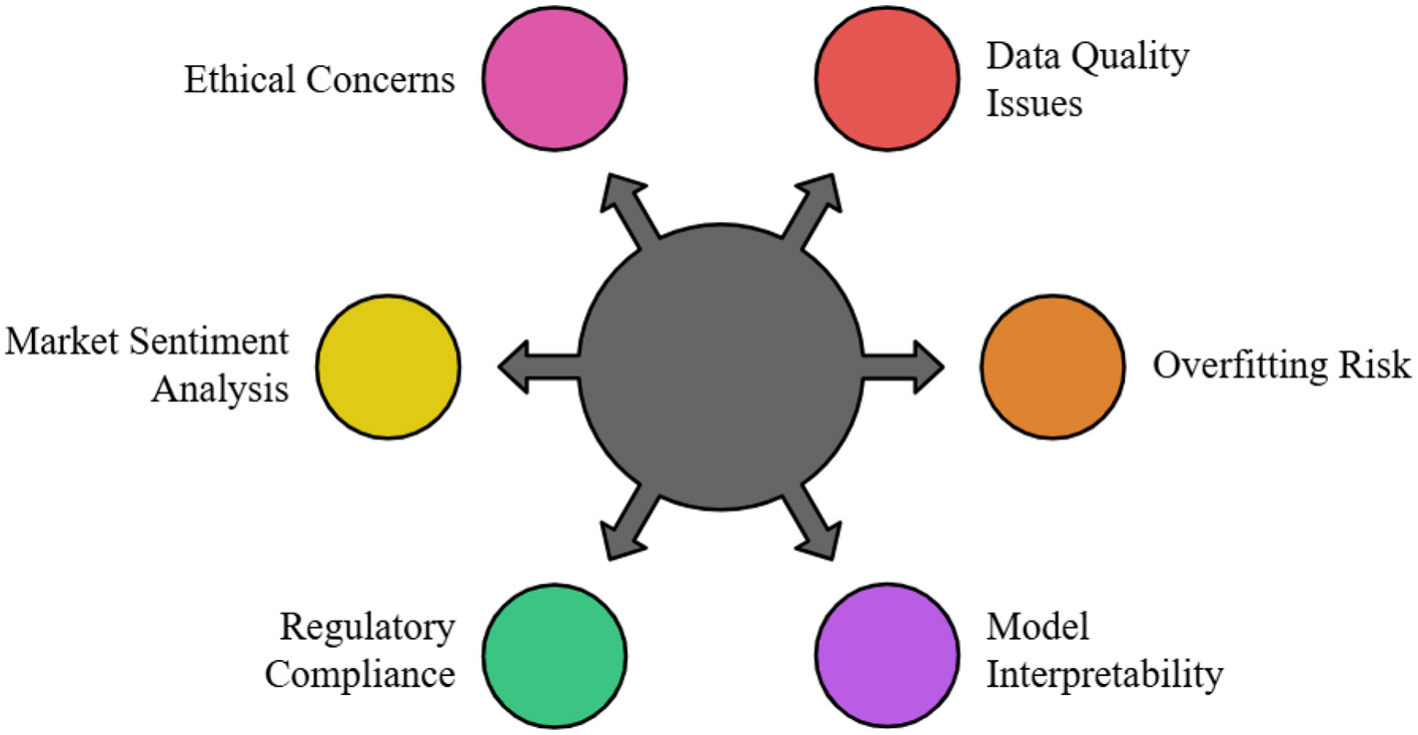
Challenges of AI and ML in stock market prediction.

One of the main factors influencing markets is consumer psychology, which is very difficult to assess. However, the markets' response to news, rumors, and other events is sometimes unexpected, making monitoring difficult when machine learning techniques are employed. Although it is one method of assessing market sentiment using publicly accessible data sources, including news stories, social media, and other sources, its effectiveness is seriously questioned. It is not easy to represent sentiment and, as a result, to incorporate it into an analytical model, even though sentiment may contribute to price swings (Chopra and Sharma, 2021; Kurani et al., 2021). Building and implementing AI models is a complex process that requires a significant investment in processing power, human resources, and technology. Compared to large companies, some small businesses may be less equipped to deploy advanced machine learning because they lack the necessary funds or a dedicated department for data scientists and big data technologies. Such an environment may lead to uneven growth in AI and machine learning in the financial sector, further exacerbating the divide between market leaders and entrants (Chhajer et al., 2021; Soni et al., 2022; Milana and Ashta, 2021). It is anticipated that the ethical concerns surrounding the use of AI applications will increase as the technology becomes more widely used in the financial sector. These issues might include, for example, fundamental algorithmic biases that lead a model to favor one group over another, potentially encouraging unequal trade between the different groups. Thus, the potential for AI to manipulate markets raises questions about accountability and control. To preserve the public's confidence and adhere to moral principles, the financial model's applications must demonstrate the institutions' fairness and openness (Zhang et al., 2023; Thakkar and Chaudhari, 2020; Mhlanga, 2023). Figure 16 illustrates the intricate challenges surrounding AI and machine learning in stock market prediction, including ethical concerns, data quality issues, overfitting risk, interpretability, and regulatory issues, as well as an example application of sentiment analysis in the market.

## 7 Future prospects

Artificial intelligence has great promise for enhancing the financial industry, particularly in stock market forecasting. Improvements in machine learning (ML) algorithms are primarily responsible for these new methods, which enhance the type and accuracy of financial forecasting (Ge, 2024). Artificial intelligence technology has a very high capability for learning and data analysis. AI and machine learning are capable of processing real-time data, including balance sheets, financial journals, news articles, tweets, matrix trading, and journal feeds, to anticipate stock market trends. This ability also involves evaluating the market to help businesses make informed investment decisions. These algorithms are pretty practical at categorizing various factors that a human study might overlook, as they are trained on patterns observed in historical data (Goel et al., 2022; Ooi et al., 2023). Machine learning algorithms are continually improving the accuracy of stock price projections, and the body of knowledge is expanding daily. Reinforcement learning and deep learning are required. From one operation to the next, some may increase their accuracy by learning and growing from new sample data. This dynamic learning capacity enables AI systems to remain stable amid market changes (Qatawneh et al., 2024; Yousef et al., 2023). Using natural language processing (NLP), AI can combine structured data—such as numbers—with unstructured data from sources like news articles and tweets to identify patterns. As a result of this integration, the machine learning system can forecast the market's mood, which, in turn, influences stock prices (Arabiat and Alshurafat, 2024). In addition to numerical values, individuals also express their opinions about specific companies or sectors, which may help investors influence the market in ways statistics cannot reveal. This makes such research valuable (Chen et al., 2022).

Artificial intelligence has been gradually changing how transactions are executed in stock exchange markets through algorithms. In terms of speed and frequency, they can perform transactions that surpass human capabilities and conduct analyses using a wide range of criteria. More sophisticated algorithms, operating in real time for sentiment analysis and prediction, will enhance trading strategies (Perifanis and Kitsios, 2023). Additionally, it is anticipated that AI will significantly alter risk management practices in the financial sector. Machine learning algorithms may identify trends that could indicate dangers and result in losses, as they are trained on data (Nti et al., 2020). The development of a diversified portfolio aligned with customers' investment goals and risk tolerance will be enabled by advanced, artificial intelligence-based portfolio management. Artificial intelligence can be used to analyze the relationships between specific assets and make informed investments that maximize returns for a given level of risk (Javed et al., 2023). The use of AI in finance not only aids forecasting but also facilitates expanded credit, enhances oversight, and enables fraud detection. A firm or organization's transactions and trade activities can be analyzed and tracked in real time using machine learning, which helps identify individuals likely to commit fraud or violate established organizational standards. Since financial rules are subject to change over time, AI can adapt to these changes while ensuring that different organizations comply (Zhou, 2022; Hoxha, 2024).

In this instance, artificial intelligence (AI) may analyze the behavior patterns of investor monitors and provide appropriate investment suggestions. In addition to offering recommendations on markets to invest in based on trends, robo-advisors, which utilize artificial intelligence, can also provide recommendations regarding the amount, style, or methods of investment tailored to the client's goals, objectives, or risk tolerance. The management of client relationships may benefit from this, making it easier to approach and potentially increasing interest in investing (Dudnik et al., 2021; Seddik et al., 2024; Waqar, 2024). The use of AI in financial services holds considerable promise, but it also raises ethical concerns, as the article highlights. As machine learning systems become increasingly widespread, several significant issues must be addressed, including algorithm bias, accountability, and explainability. Maintaining dominance restrictions on AI use requires collaboration between engineers and banking-sector authorities. Notably, to increase market trust, society needs to establish moral guidelines for the use of AI in financial markets (Nguyen and Kravets, 2023; Samala et al., 2024). Therefore, it is anticipated that the level of employable skills will change as financial institutions increasingly use AI technology. As the future will see highly technologically sophisticated sectors such as data science and machine learning, there will be more job opportunities spanning both the financial and technical sectors (Espina-Romero et al., 2023). Since new positions may emerge and certain existing functions may become less critical, it will be necessary to concentrate on retraining and reskilling employees (Dadiyala and Welekar, 2024). According to a trend analysis, the financial industry can improve efficiency and dynamism by integrating artificial intelligence into stock market forecasting. Artificial intelligence trading is an example of new-generation services that might enhance market participation and accessibility, a trend becoming increasingly popular in financial markets. Given the advancements in computer use in the financial sector, it is anticipated that artificial intelligence (AI) will be introduced more frequently, resulting in favorable improvements that will enhance the standing of investors, institutions, and the economy (Ofosu-Ampong, 2024; Kumar et al., 2023; Chen C. et al., 2023; Dixit and Soni, 2023). Recent advancements in machine learning have enabled new possibilities in financial market prediction, particularly in stock price forecasting (Noviandy et al., 2024). These include reinforcement learning to develop an adaptive trading strategy, deep learning systems such as LSTMs and transformers that are better at modeling time series with varying temporal dependencies, and more straightforward machine learning, known as hybrids, which combine sentiment, technical, and fundamental analysis to improve prediction accuracy (Aiche et al., 2024). These developments incorporate additional data sources, such as social media, news, and satellite imagery, as well as explainable AI techniques to simulate transparency and transfer learning to implement the model in various markets, thereby producing more precise, flexible, and comprehensible forecasting in ever-changing financial environments (Thakkar and Chaudhari, 2020).

**Figure 17:**
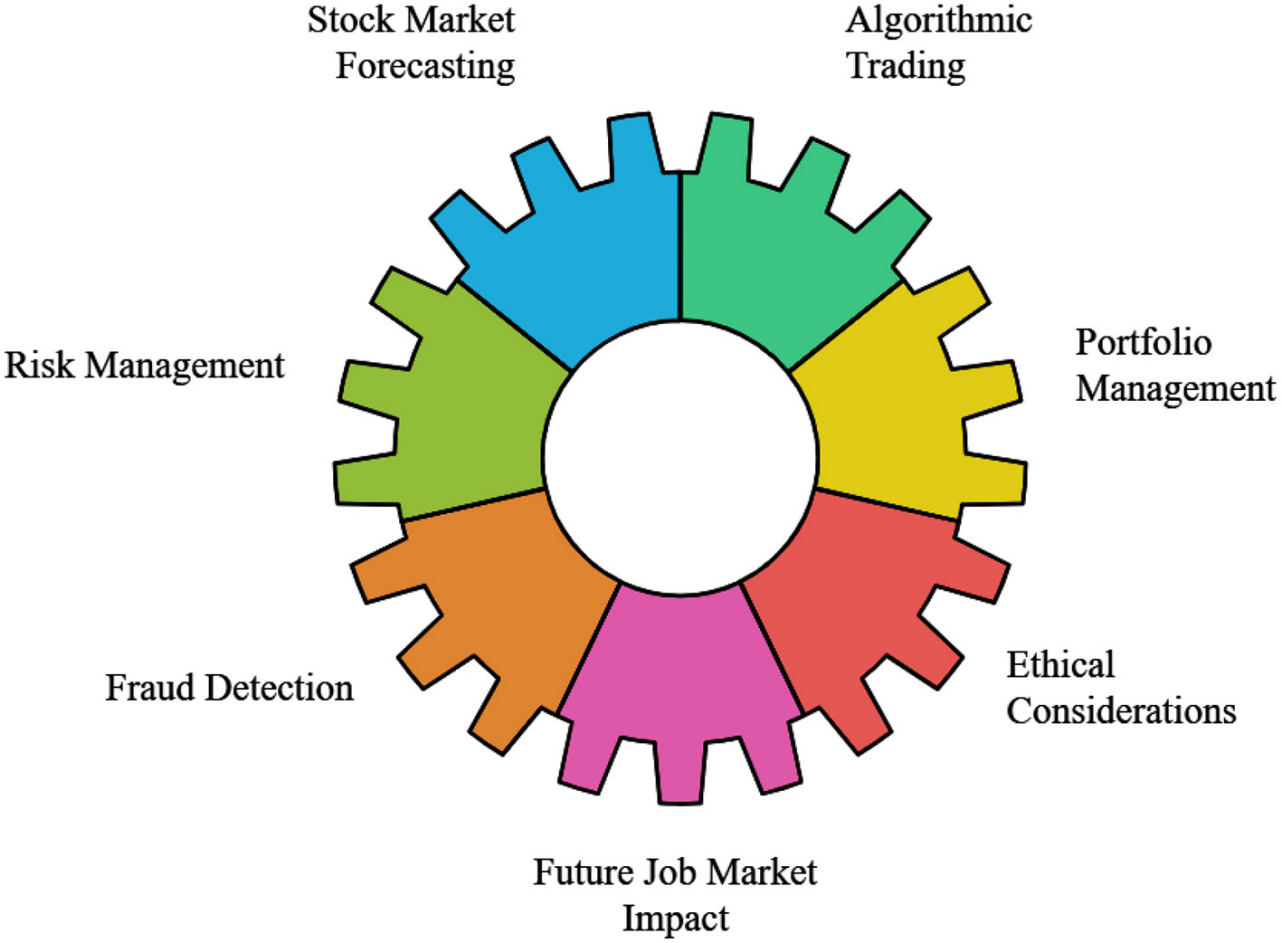
Future prospects of AI and ML in stock market prediction.

In Figure 17, the future of AI and ML in stock market prediction is shown as a cogwheel, with each application connected to support growth. To demonstrate how success in one area enhances the overall performance and efficiency of AI/ML in shaping the future of financial markets, each cog forms a semicircle, with labels for Stock Market Forecasting, Algorithmic Trading, Portfolio Management, Ethical Issues, Job Market Impact, Fraud Detection, and Risk Management.

## 8 Conclusion

Artificial Intelligence and Machine Learning have significantly improved stock price forecasting, transforming traditional techniques. This review explores the evolution of financial market analysis, highlighting the role of technology in modern finance, including machine learning systems and predictive models. Integrating fundamental, sentiment, and technical analysis into AI-based models yields holistic predictions. Hybrid models, which combine deep neural networks and prediction rule ensembles, effectively capture complex market patterns. The integration of quantum computing and blockchain technologies further enhances prediction accuracy, transparency, and financial asset management.

Data collection and purification are crucial for predictive models, as the effectiveness of AI algorithms depends on the quality of the input data. Reinforcement learning (RL) is a promising approach for real-time predictions. Transparency in financial decision-making is essential for stakeholders to trust predictions. Time series analysis, integrated with AI, improves stock price prediction and market trends modeling. These advancements provide more accurate predictions, enhanced portfolio management, and increased financial market efficiency. NLP technology helps capture market sentiment, opening a new door to high-frequency trading (HFT) within the FinTech model. The formation of patterns and trends in the market can be easily identified through quantum computing, which helps to purify data and eliminate duplicate patterns.

A key component of stock price forecasting, time series analysis remains essential for understanding market fluctuations. Market forecasting has enormous potential to improve in accuracy, efficiency, and responsiveness to shifting situations as more complex AI systems are initiated simultaneously. Moreover, ethical concerns, market sentiment analysis, overfitting risk, regulatory issues, and data quality remain significant challenges in stock market prediction using machine learning. Additionally, topics such as data quality, market volatility, and model interpretability persist in this system. However, fraud detection, risk management, and portfolio governance create a new horizon of possibilities in this sector, which opens up new scenarios in the job market. The potential of AI and ML technologies to enhance financial prediction systems is poised to revolutionize market analysis and forecasting, paving the way for more informed financial decision-making and more strategic, thoughtful investment strategies.

AI-driven forecasting has achieved significant progress, yet several research gaps remain unresolved. Future studies should prioritize interpretable, regulation-compliant models that meet transparency requirements set by global financial authorities. Robustness to market regime shifts, adversarial risks, and data inconsistencies must be improved for real-world deployment. Although quantum computing offers long-term promise, its current limitations restrict immediate application. Ethical governance frameworks and standardized evaluation protocols are essential for safe adoption. Addressing these challenges will enable AI-based forecasting models to be integrated more reliably within financial institutions.
